# Developmental transcriptomic analyses for mechanistic insights into critical pathways involved in embryogenesis of pelagic mahi-mahi (*Coryphaena hippurus*)

**DOI:** 10.1371/journal.pone.0180454

**Published:** 2017-07-10

**Authors:** Elvis Genbo Xu, Edward M. Mager, Martin Grosell, John D. Stieglitz, E. Starr Hazard, Gary Hardiman, Daniel Schlenk

**Affiliations:** 1 Department of Environmental Sciences, University of California, Riverside, California, United States of America; 2 Department of Biological Sciences, University of North Texas, Denton, Texas, United States of America; 3 Department of Marine Biology and Ecology, University of Miami, Miami, Florida, United Sates of America; 4 Center for Genomic Medicine, Medical University of South Carolina, Charleston, South Carolina, United Sates of America; 5 Computational Biology Resource Center, Medical University of South Carolina, Charleston, South Carolina, United Sates of America; 6 Departments of Medicine & Public Health Sciences, Medical University of South Carolina, Charleston, South Carolina, United Sates of America; 7 Laboratory for Marine Systems Biology, Hollings Marine Laboratory, Charleston, South Carolina, United Sates of America; Laboratoire Arago, FRANCE

## Abstract

Mahi-mahi (*Coryphaena hippurus*) is a commercially and ecologically important species of fish occurring in tropical and temperate waters worldwide. Understanding early life events is crucial for predicting effects of environmental stress, which is largely restricted by a lack of genetic resources regarding expression of early developmental genes and regulation of pathways. The need for anchoring developmental stages to transcriptional activities is highlighted by increasing evidence on the impacts of recurrent worldwide oil spills in this sensitive species during early development. By means of high throughput sequencing, we characterized the developmental transcriptome of mahi-mahi at three critical developmental stages, from pharyngula embryonic stage (24 hpf) to 48 hpf yolk-sac larva (transition 1), and to 96 hpf free-swimming larva (transition 2). With comparative analysis by multiple bioinformatic tools, a larger number of significantly altered genes and more diverse gene ontology terms were observed during transition 2 than transition 1. Cellular and tissue development terms were more significantly enriched in transition 1, while metabolism related terms were more enriched in transition 2, indicating a switch progressing from general embryonic development to metabolism during the two transitions. Special focus was given on the most significant common canonical pathways (e.g. calcium signaling, glutamate receptor signaling, cAMP response element-binding protein signaling, cardiac β-adrenergic signaling, etc.) and expression of developmental genes (e.g. collagens, myosin, notch, glutamate metabotropic receptor etc.), which were associated with morphological changes of nervous, muscular, and cardiovascular system. These data will provide an important basis for understanding embryonic development and identifying molecular mechanisms of abnormal development in fish species.

## Introduction

Spatial and temporal dynamics of patterns of gene expression during development provides important insights into mechanisms linking genotype with phenotype [1]. The lack of genetic resources and tools largely restricts our investigation on normal development in non-model organisms [2, 3]. Next-generation DNA and RNA sequencing in combination with advanced bioinformatics are gradually becoming more affordable and have the potential to anchor molecular events to developmental processes in non-model species. Understanding the early development of susceptible non-model fish is a prerequisite for predicting impacts of contaminants or environmental stress.

In particular, the Deepwater Horizon (DWH) incident in 2010, the largest marine oil spill in U.S. history, resulted in exposure of embryonic and larval life stages of pelagic fish species [4–6]. Numerous studies have documented that fish embryos and larvae were very sensitive to crude oil toxicity, and identified a variety of abnormalities in cardiac function, oxygen consumption, kidney development, craniofacial morphology (eye and jaw), the nervous system, as well as reduced swimming performance and population [7–14]. Our previous study demonstrated that crude oil exposure resulted in altered regulation of genes involved in metabolism, steroid biosynthesis, cardiac development, and vision [9]. However, since previous studies only evaluated differential expression of the transcriptome comparing oil-treated animals with controls, it is necessary to characterize molecular pathways of normal developmental stages to better understand the impacts of oil.

In the current study, a set of raw sequencing reads of normal developing embryonic mahi-mahi generated in our previous study [9] were processed and analyzed for three distinct embryonic stages, including the pharyngula embryonic stage (24 hpf), the yolk-sac larva stage (48 hpf), and the free-swimming larva stage (96 hpf) [15]. To better understand the molecular signaling associated with each stage, the transcriptional profiles in these two critical developmental transitions were determined. Measurements of activated canonical pathways along with identification of corresponding genes were interfaced with advanced comparative bioinformatics tools to link molecular events to morphological endpoints. The data will provide an important genetic resource for understanding the developmental mechanisms of the embryonic, yolk-sac, and free-swimming larval stages of mahi-mahi, and provide baseline data for assessing the development of embryos under environmental stress.

## Materials and methods

### Animals

Mahi-mahi (*Coryphaena hippurus*) broodstock were caught off the coast of South Florida (25° 40’N, 80° 00’N) using hook and line angling techniques and then directly transferred to University of Miami Experimental Hatchery (UMEH). Field collection permit (Special Activity License—#SAL-16-0932C-ABC) is issued by the Florida Fish and Wildlife Conservation Commission to Dr. Daniel Benetti at the University of Miami—RSMAS. Broodstock were acclimated in 80 m^3^ fiberglass maturation tanks equipped with recirculating and temperature controlled water. All embryos used in the experiments described here were collected within 2–10 h following a volitional (non-induced) spawn using standard UMEH methods [16, 17]. Three replicates were used per time point with 25 embryos per replicate. All animal experiments were performed ethically and in accordance with Institutional Animal Care and Use Committee (IACUC protocol number 15–019) approved by the University of Miami IACUC committee, and the institutional assurance number is A-3224-01.

### Imaging

Embryos or larvae were collected from each replicate beaker and imaged to characterize developmental features at 24, 48 and 96 hpf. Embryos or larvae were imaged using either a Fire-i400 or Fire-i530c digital camera (Unibrain, San Ramon, CA) mounted on a Nikon SMZ800 stereomicroscope. The sample size of randomly imaged individuals was 60, 42, and 47 at 24 hpf, 48 hpf and 96 hpf, respectively. Images were collected using iMovie software and calibrated using a stage micrometer. Embryos and larvae were staged and characterized according to Mito [15].

### RNA isolation, cDNA library construction and sequencing

The surviving embryos or larvae from each replicate were pooled and RNA was isolated and purified with RNeasy Mini Kit (Qiagen, Valencia, California). The total RNA sample was quantified by NanoDrop ND-1000 Spectrophotometer (Nanodrop Technologies, Wilmington, DE, USA). 200 ng of total RNA was used to prepare RNA-Seq libraries using the TruSeq RNA Sample Prep kit following the protocol described by the manufacturer (Illumina, San Diego, CA). Single Read 1X50 sequencing was performed on each of the triplicate samples using Illumina HiSeq 2500 at the Center for Genomics Medicine, Medical University of South Carolina, Charleston, SC, with each individual sample sequenced to a minimum depth of ~50 million reads. Data were subjected to Illumina quality control (QC) procedures (>80% of the data yielded a Phred score of 30). A detailed description of these methods is presented in Xu et al. (2016). The read data for the samples was deposited in the NCBI database (Accession Number: GSM2100982 to GSM2100995).

### A reference-transcriptome-guided analysis by OnRamp

Raw reads processing and annotation analysis was carried out on an OnRamp Bioinformatics Genomics Research Platform (OnRamp Bioinformatics, San Diego, CA) as previously described [9]. OnRamp’s advanced Genomics Analysis Engine utilized an automated RNAseq workflow to map read alignment to the *Takifugu rubripes* transcriptome (FUGU4) using BLASTX: Basic Local Alignment Search Tool, and generate gene-level count data. Differential analysis of count data and PCA analysis was carried out using the DESeq2 package (https://www.bioconductor.org/packages/release/bioc/html/DESeq2.html). The PCA plot was customized using ggplot2 (version 2.2.1, http://cran.fhcrc.org/web/packages/ggplot2/index.html). The Fugu transcriptome was chosen as reference over zebrafish (*Danio rerio*) model, because Tetraodontiformes (Fugu) and Perciformes (*Coryphaena hippurus*, mahi-mahi) are close phylogenetic relatives, while Cypriniformes (zebrafish) are a distant phylogenetic relative to mahi-mahi [18]. Transcript count data from DESeq2 analysis of the samples were sorted according to their false discovery rate (FDR) at which a transcript is called significant. The protein FASTA sequences from Ensembl for Fugu were compared using Ensembl's homology to create protein FASTA files that contained a human Entrez gene ID that mapped via Fugu to Mahi-mahi. A more detailed description of the commercial OnRamp platform can be found elsewhere [19] and details on the pipeline utilized can be found in Xu et al. [9].

### Gene ontology and Ingenuity Pathway Analyses

The sorted transcript list of mahi-mahi generated by OnRamp was mapped to human orthologs to generate HGNC (HUGO Gene Nomenclature Committee) gene symbols for downstream gene ontology (GO) term analysis, using ToppGene Suite [20]. This approach has been demonstrated to improve functional analysis of fish genes with a more sensitive systems level interrogation, by providing access to the best-annotated databases and tools for human/mouse/rat models, despite limitations of the mapping due to the extra genome duplication events in teleost fish and species differences in gene function and pathways [9, 21, 22]. GO terms for molecular function, molecular component, biological process, pathway and phenotype were considered significantly enriched when p < 0.05. The enriched GO terms were visualized by Gorilla [23, 24].

Comparative analysis on biological function and canonical pathway was further conducted. If individual genes are not commonly regulated between methods/conditions, commonality may still exist on the level of pathway regulation. Comparative pathway analysis could also provide additional information on modes of action of toxicants. Ingenuity Pathway Analyses (IPA) (Ingenuity Systems Inc., Redwood City, CA, USA) was used to compare at different developmental transitions to identify similarities and differences in biological functions and canonical pathways (IPA-Comparison analysis). The rationale of using IPA is that it utilizes expertly curated biological interactions and functional annotations from multiple databases. Each modeled relationship between biological molecules, functions and pathways has been reviewed by experienced bioinformaticians as well as biologists. IPA significantly improves our understanding on the connections and interactions among genes, pathway, functions, and development characteristics. Fisher’s exact test was used to calculate a p-value determining the probability that the association between the genes in the dataset and the pathways as opposed to this occurring by chance alone.

## Results

### Embryonic morphological metrics during development

**Fig 1:**
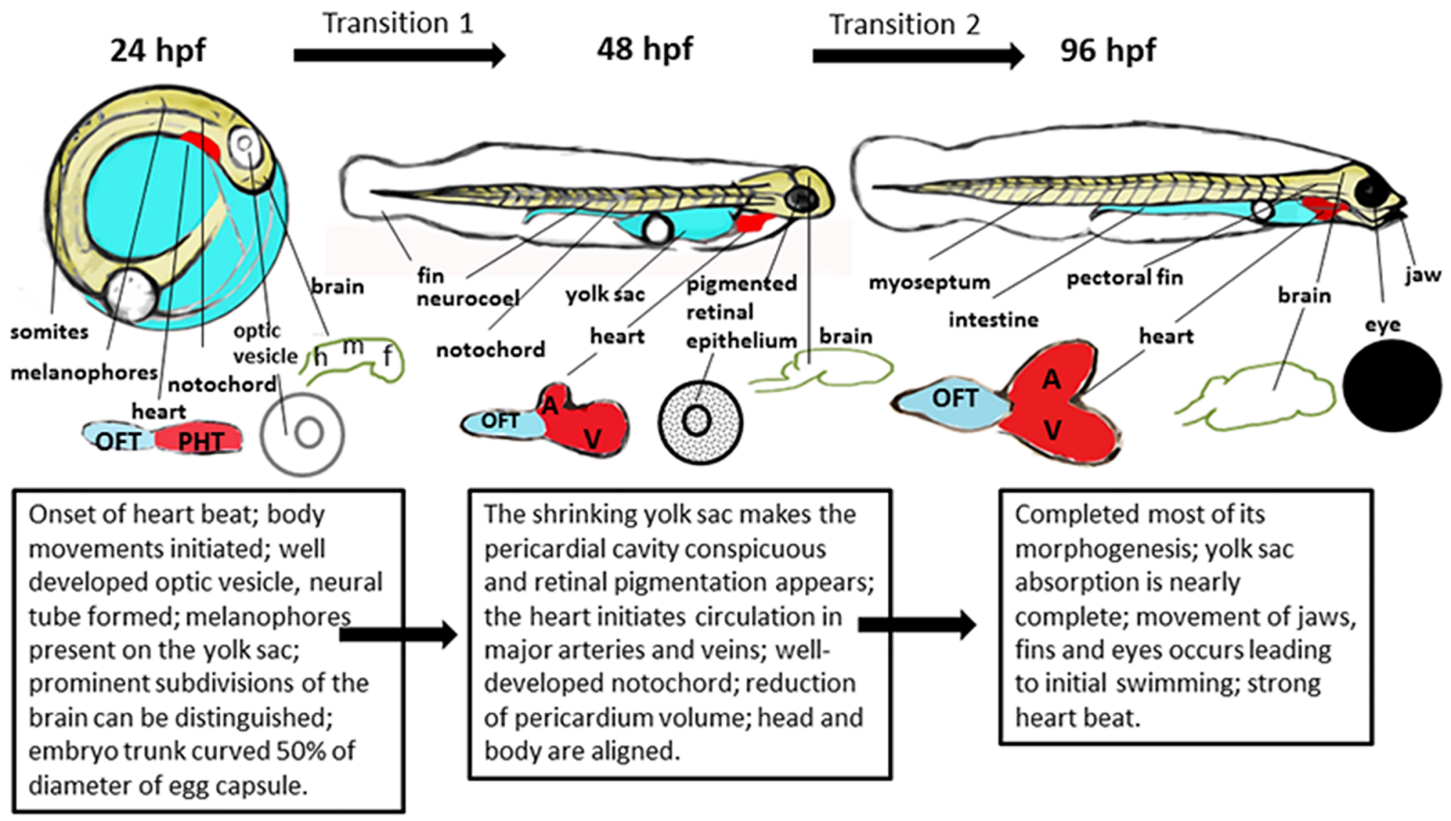
The development of the pharyngula (24 hpf) to yolk sac larva (48 hpf) and the swimming larva (96 hpf). OFT, outflow tract; PHT, primitive heart tube; A, atria; V, ventricle; F, forebrain; M, midbrain; H, hindbrain.

The development of the sampled embryos and larvae were highly synchronized. At 24 hpf, the heart of the embryo begins to beat and body movements are initiated. The optic vesicle is well developed, neural tube is formed and melanophores are present on the yolk sac. Prominent subdivisions of the brain can be distinguished, while the brain area is still hollow. Hollowing from the neural rod into the neural tube is also nearly complete. A linear heart tube is also formed. At transition 1 during 48 hpf, the shrinking yolk sac makes the pericardial cavity conspicuous and retinal pigmentation appears. The ventricle and atrium become morphologically distinguishable, and the notochord is well-developed. The brain is sculptured into five lobes (telencephalon, diencephalon, mesencephalon, metencephalon, and myelencephalon). During transition 2 to 96 hpf, the swimming larva has completed most of its morphogenesis and yolk sac absorption is nearly complete. The cardiac ventricle grows in similar size to the atrium. Movement of jaws, fins and eyes occurs leading to initial swimming. Fig 1 depicts the pharyngula, the yolk sac larva, and the swimming larva.

### Sequencing and differentially expressed genes (DEGs)

**Fig 2:**
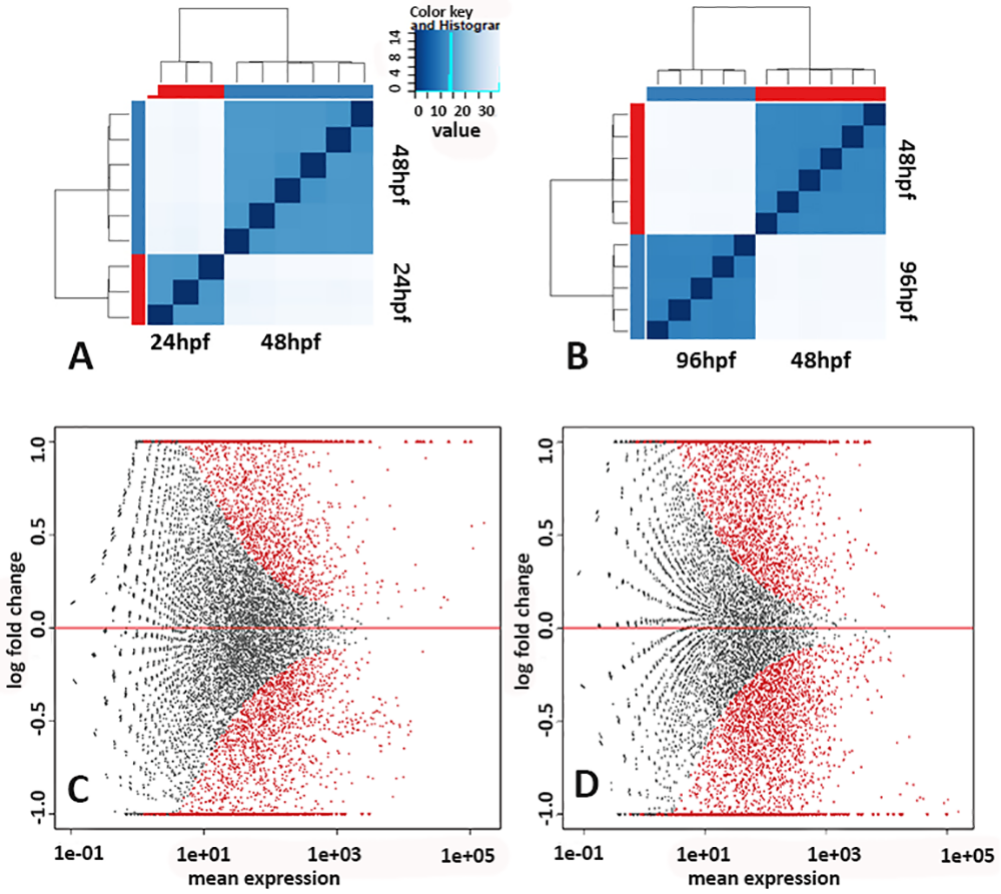
Heatmaps (A and B) showing the Euclidean distances between the samples as calculated from the DEseq2 variance stabilizing transformation of the count data. Samples are clustered by similarity. The samples from each developmental time point clustered together indicating global differences between the different stages. Plot of normalized mean counts (expression) versus log2 fold change for comparisons in transition 1 (24 hpf versa 48 hpf; C) and transition 2 (48 hpf versa 96 hpf; D). The X-axis plots normalized mean expression and the Y-axis is the log2 fold change (FC). Black dots represent non-significant genes, whereas red dots indicate significant differentially expressed genes (q < 0.05).

**Fig 3:**
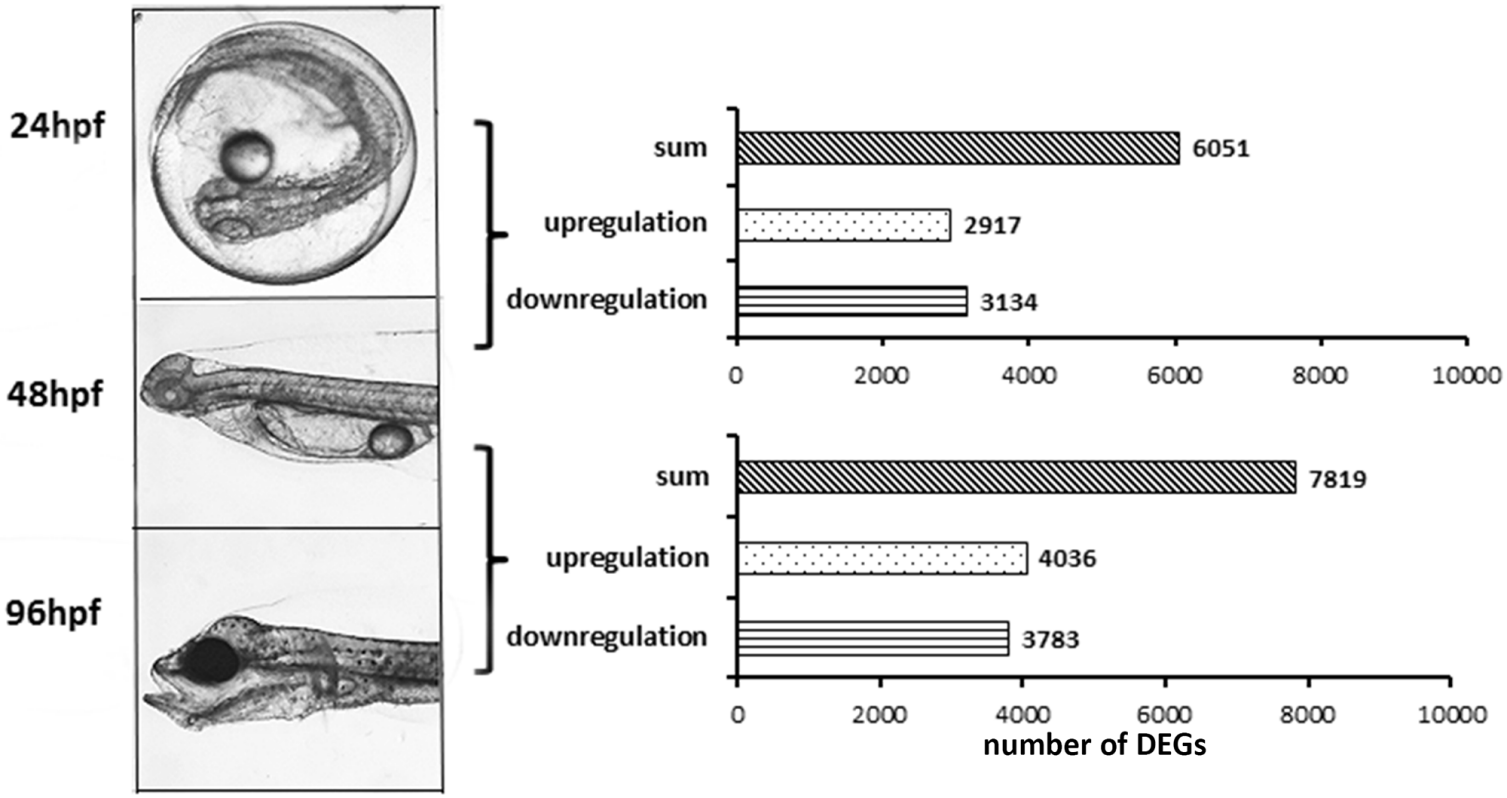
Developmental stages and numbers of differentially expressed genes (DEGs). Number of DEGs in the two transitions between the three stages of development, from 24 hpf (pharyngula) to 48 hpf (yolk-sac larva) to 96 hpf (free-swimming larva), with bars representing numbers of total, upregulated and downregulated DEGs. Adjusted p-value < 0.1. The X-axis plots the numbers of DEGs, and the Y-axis represents total, upregulated and downregulated DEGs.

Analysis of RNA-seq data resulted in approximately 711 million reads, with 1.7 million hits against Fugu protein sequences in each sample (Table 1). The samples collected from the three different developmental stages clustered separately from each group, indicating global transcriptomic differences among the three stages (Fig 2a and 2b). PCA analysis and the identity plot indicated a good separation between the 24, 48 and 96 hpf time points, and small divergences between individual samples (S1 Fig). The representing differentially expressed genes (DEGs) in the two transitions were visually increasing with progressing development (Fig 2c and 2d). The number of significantly up- and down- regulated DEGs were 2,917 and 3,134 in transition 1 (24 to 48 hpf), and 4,036 and 3,783 in transition 2 (48 to 96 hpf), respectively (Fig 3). In transition 1, the significantly expressed genes with the largest fold change included adenosine monophosphate deaminase (*ampd1*), muscle phosphorylase (*pygm*), sarcalumenin (*srl*), neurotransmitter transporter (*slc6a8*), collagens, myosin, and myosin binding protein (*mybpc2*). In transition 2, the most significantly expressed genes were adenylosuccinate synthase (*addssl1*), aldehyde dehydrogenase (*aldh1*), amylase (*amy*), ATPase, guanine nucleotide binding proteins, myosin, notch, and retinal G protein coupled receptor (*rgr*). These genes were scattered across diverse functions and processes. In order to identify systems-level function and compare the functions from different stages and, the DEGs were furthered analyzed for Gene Ontology (GO) terms in the below section.

**Table 1:** Statistics of sequencing and mapping to *Takifugu rubripes* transcriptome (FUGU4).

Samples (n = 3)	Mean Seqs in FASTQ File	Hits against Fugu protein sequences
24 hpf	49,538,883	1,605,640
48 hpf	50,136,278	1,759,212
96 hpf	52,282,330	1,776,690
median	51,466,400	1,757,246
Total	710,845,971	24,255,645

### Gene ontology (GO) functional terms analysis

We linked DEGs to GO functional terms by ToppGene in order to identify their system-level functions. The significantly enriched ontology of the two developmental transitions showed a higher degree of similarity in molecular function and cellular components, but different in biological process and pathways. More diverse GO terms were consistently enriched in all molecular function (S2 Fig), cellular component (S3 Fig) and biological process (S4 Fig) categories during transition 2 over transition 1. Within all the shared GO terms, more genes were involved in transition 2 than in transition 1 (Table 2). Molecular function was very similar between transition 1 and transition 2, dominated by “binding” and kinase activity, including macromolecular complex binding, nucleotide binding, ATP binding, enzyme binding, RNA binding, protein complex binding. Synapse, neuron projection, cell junction, dendrite, and somatodendritic compartment were the predominant cellular components enriched during both transitions. Catalytic complex and transferase complex topped the cellular component list of only transition 2. The most representative pathways during both developmental transitions included metabolism, neuronal system, transmission across chemical synapses, signaling by Wnt and focal adhesion, whereas significant numbers of genes were altered in cell cycle, TGF-beta Receptor Signaling Pathway and splice-related terms in only transition 2, and extracellular matrix (ECM)-related terms only in transition 1. Consistent with enriched cellular component and pathway terms, the top biological processes in both transitions were neurogenesis, generation of neurons, neuron differentiation, neuron development, cell morphogenesis involved in differentiation, response to endogenous stimulus, regulation of cell development and organ morphogenesis. Compared to transition 1, over 1,000 genes were involved in cellular catabolic processes, organic substance catabolic processes, positive regulation of RNA metabolism, nitrogen compound metabolism, and positive regulation of transcription only during transition 2. Taken together the significantly enriched GO terms indicate that there is a critical shift in pathways taking place from general embryonic development to metabolism during transition 1 to transition 2.

**Table 2:** Top enriched molecular functions, components and biological processes. The shared terms between the two transitions are highlighted in bold. *p*-value method, hypergeometric probability mass function with FDR correction.

*Transition 1 (24–48 hpf)*			*Transition 2 (48–96 hpf)*		
***Molecular Functions***	***p_FDR***	***Genes***	***Molecular Functions***	***p_FDR***	***Genes***
**poly(A) RNA binding**	1.64E-47	621	**macromolecular complex binding**	5.64E-40	938
**RNA binding**	4.29E-39	776	**adenyl ribonucleotide binding**	1.40E-33	864
**macromolecular complex binding**	3.02E-36	764	**ribonucleotide binding**	1.90E-33	1036
**adenyl nucleotide binding**	5.85E-27	695	**adenyl nucleotide binding**	2.41E-33	868
**purine nucleotide binding**	7.17E-27	825	**purine ribonucleotide binding**	2.78E-33	1027
**ribonucleotide binding**	1.18E-26	824	**purine nucleotide binding**	4.59E-33	1033
**purine ribonucleotide binding**	1.18E-26	818	**nucleoside binding**	6.83E-32	1009
**nucleoside binding**	1.23E-26	808	**ATP binding**	9.93E-32	835
**adenyl ribonucleotide binding**	1.23E-26	688	**ribonucleoside binding**	1.50E-31	1004
**ribonucleoside binding**	1.28E-26	805	**purine nucleoside binding**	2.49E-31	1003
**purine ribonucleoside binding**	1.82E-26	803	**enzyme binding**	2.50E-31	1049
**purine nucleoside binding**	1.82E-26	804	**purine ribonucleoside binding**	2.96E-31	1001
**purine ribonucleoside triphosphate**	1.82E-26	798	**purine ribonucleoside triphosphate**	8.48E-31	993
**ATP binding**	8.17E-26	668	**poly(A) RNA binding**	5.09E-29	683
**enzyme binding**	1.43E-21	819	chromatin binding	6.16E-26	336
**protein complex binding**	3.47E-21	497	**RNA binding**	2.34E-22	873
transition metal ion binding	2.98E-20	636	**protein complex binding**	4.47E-21	602
zinc ion binding	3.03E-18	535	phosphotransferase activity, alcohol group acceptor	1.72E-18	452
transferase activity	1.63E-15	451	gated channel activity	1.82E-18	222
**kinase activity**	4.45E-15	391	**kinase activity**	1.82E-18	487
***Biological Processes***	***p_FDR***	***Genes***	***Biological Processes***	***p_FDR***	***Genes***
**neurogenesis**	9.41E-64	847	**generation of neurons**	1.16E-63	959
**generation of neurons**	3.65E-61	799	**neurogenesis**	3.54E-63	1008
**neuron differentiation**	9.86E-61	744	**neuron differentiation**	1.05E-60	884
**neuron development**	2.55E-46	591	**neuron development**	4.20E-44	697
regulation of nervous system development	3.62E-46	492	embryo development	1.01E-42	704
**cell morphogenesis involved in differentiation**	3.47E-45	472	cellular catabolic process	6.82E-42	1045
neuron projection development	3.03E-44	518	**cell morphogenesis involved in differentiation**	2.35E-40	545
**regulation of cell development**	1.12E-43	530	organic substance catabolic process	1.83E-39	1076
neuron projection morphogenesis	3.90E-42	373	**response to endogenous stimulus**	6.85E-39	992
**regulation of multicellular organism**	2.60E-41	885	**organ morphogenesis**	1.62E-38	685
regulation of neurogenesis	1.26E-39	435	positive regulation of RNA metabolic process	7.37E-38	917
regulation of cell differentiation	3.43E-38	800	positive regulation of nitrogen compound metabolic process	1.51E-37	1085
cell morphogenesis involved in neuron rentiation	1.05E-37	346	positive regulation of nucleobase-containing compound metabolic process	1.25E-36	1028
central nervous system development	1.87E-37	521	neuron projection development	1.25E-36	594
regulation of neuron differentiation	2.01E-37	374	positive regulation of RNA biosynthetic process	4.21E-36	888
**response to endogenous stimulus**	5.65E-36	808	regulation of transcription from RNA polymerase II	1.57E-35	1064
**organ morphogenesis**	3.67E-35	563	positive regulation of transcription, DNA-templated	6.96E-35	875
head development	1.30E-33	428	positive regulation of nucleic acid-templated transcription	6.96E-35	875
regulation of anatomical structure	3.56E-33	560	**regulation of multicellular organism**	9.16E-35	1050
axon development	7.06E-33	302	**regulation of cell development**	2.28E-34	604
***Cellular component***	***p_FDR***	***Genes***	***Cellular component***	***p_FDR***	***Genes***
**synapse**	1.25E-51	493	**synapse**	1.28E-55	586
**neuron part**	1.89E-50	766	**neuron part**	5.08E-54	927
**neuron projection**	1.89E-50	625	**neuron projection**	4.22E-48	736
**synapse part**	4.26E-44	406	**cell junction**	7.21E-46	733
**cell junction**	8.47E-44	610	**synapse part**	1.07E-43	474
**dendrite**	7.65E-34	333	catalytic complex	4.32E-42	661
**somatodendritic compartment**	4.19E-33	447	transferase complex	2.43E-35	463
**postsynapse**	1.93E-28	260	**dendrite**	8.89E-33	388
**cell-substrate junction**	3.41E-28	239	**membrane region**	2.39E-30	720
cell-substrate adherens junction	7.60E-28	236	**somatodendritic compartment**	2.92E-29	521
**focal adhesion**	1.71E-27	233	plasma membrane region	2.17E-27	601
**axon**	2.49E-27	308	nucleoplasm part	2.19E-27	448
endoplasmic reticulum	7.45E-26	745	**axon**	8.92E-27	362
adherens junction	7.08E-25	267	**postsynapse**	2.16E-26	299
anchoring junction	1.94E-24	274	presynapse	9.87E-26	239
intracellular ribonucleoprotein complex	2.59E-24	373	**synaptic membrane**	2.22E-22	209
ribonucleoprotein complex	2.59E-24	373	cell projection part	1.17E-21	628
cell body	3.74E-23	348	**cell-substrate junction**	8.54E-20	260
**synaptic membrane**	2.21E-22	180	**focal adhesion**	1.69E-19	254
**membrane region**	1.61E-20	561	transmembrane transporter complex	1.69E-19	219
***Pathways***	***p_FDR***	***Genes***	***Pathways***	***p_FDR***	***Genes***
**Developmental Biology**	1.88E-11	223	**Metabolism**	3.00E-22	891
Metabolism of proteins	7.40E-11	333	**Neuronal System**	3.86E-21	216
**Neuronal System**	9.61E-10	162	**Transmission across Chemical Synapses**	1.01E-15	151
Extracellular matrix organization	4.85E-09	147	**Developmental Biology**	3.17E-15	274
Axon guidance	9.13E-09	145	Processing of Capped Intron-Containing Pre-mRNA	2.55E-11	109
**Transmission across Chemical Synapses**	9.13E-09	117	Neurotransmitter Receptor Binding And Downstream Transmission In The Postsynaptic Cell	1.95E-10	108
Ribosome	1.16E-08	86	**Metabolism of amino acids**	3.77E-10	136
**Metabolism**	4.88E-08	660	Spliceosome	9.75E-09	97
**Focal adhesion**	4.97E-07	115	Cell Cycle, Mitotic	3.34E-08	251
Collagen formation	4.97E-07	59	**Signaling by Wnt**	4.18E-08	125
Ensemble of genes encoding core ECM	5.17E-07	145	mRNA Splicing	5.81E-08	84
Axon guidance	8.62E-07	78	mRNA Splicing—Major Pathway	5.81E-08	84
Biosynthesis of amino acids	1.29E-06	51	mRNA processing	6.61E-08	98
**Signaling by Wnt**	2.63E-06	102	TGF-beta Receptor Signaling Pathway	9.57E-08	108
ECM-receptor interaction	2.63E-06	57	Carbon metabolism	9.70E-08	72
Translation	2.63E-06	113	**Focal adhesion**	1.01E-07	137
Proteoglycans	1.08E-05	119	M Phase	1.15E-07	151
**Metabolism of amino acids**	1.08E-05	105	Wnt Signaling Pathway NetPath	1.15E-07	85

### Ingenuity Pathway Analyses (IPA)

**Fig 4:**
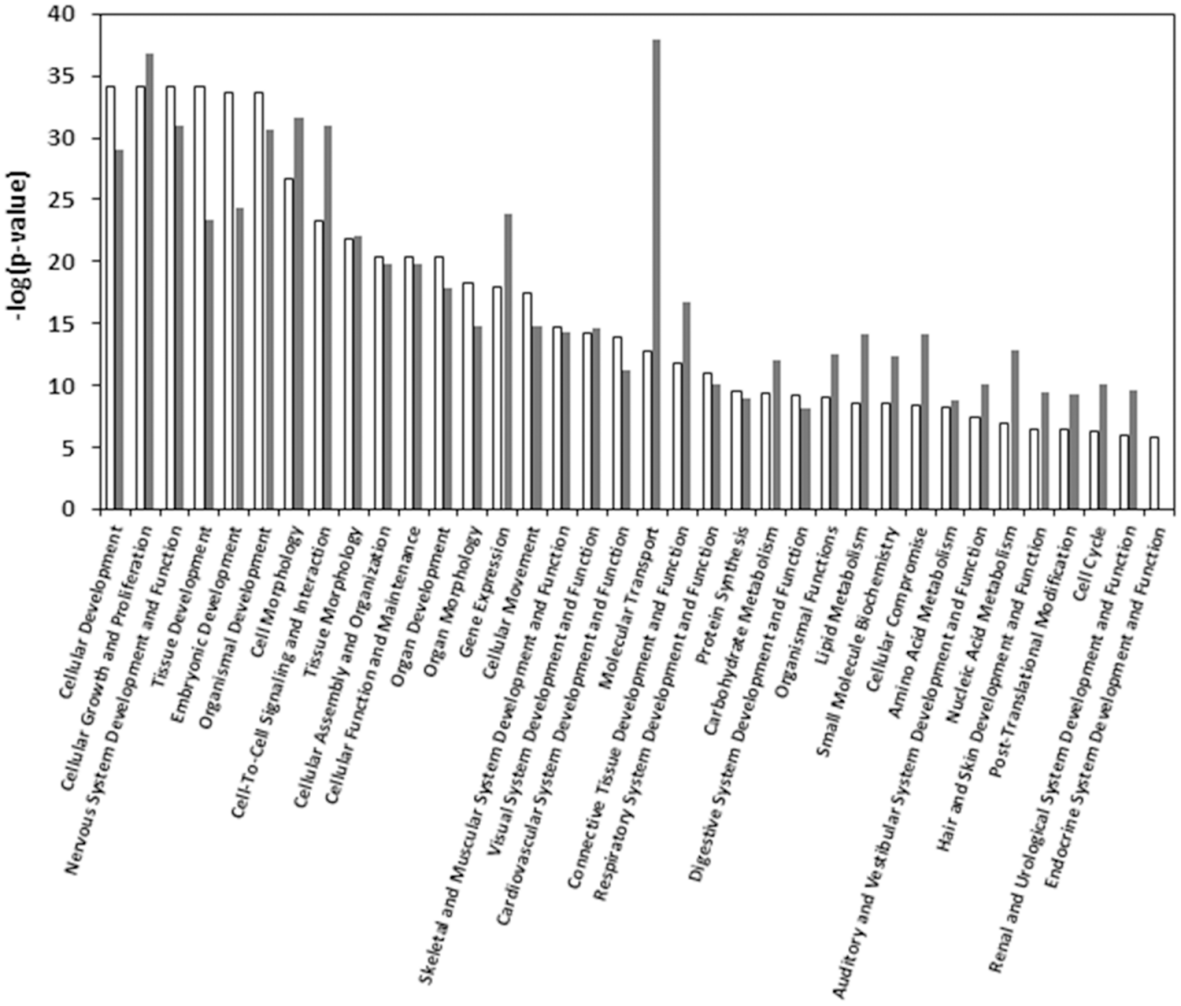
Bar chart representing the most significant biological functions involved in transition 1 (white bar) and transition 2 (gray bar). The y-axis displays the negative log significance by Fisher’s Exact Test. The p-value is a measure of the likelihood that the association between a set of genes in the analyzed dataset and a related function is due to random association. The smaller the p-value, the less likely that the association is random and the more significant the association. In general, p-values < 0.05 (-log = 1.3) indicate a statistically significant, non-random association. Functions are listed from most to least significant. A -log (p-value) cutoff was set to 1.3.

**Fig 5:**
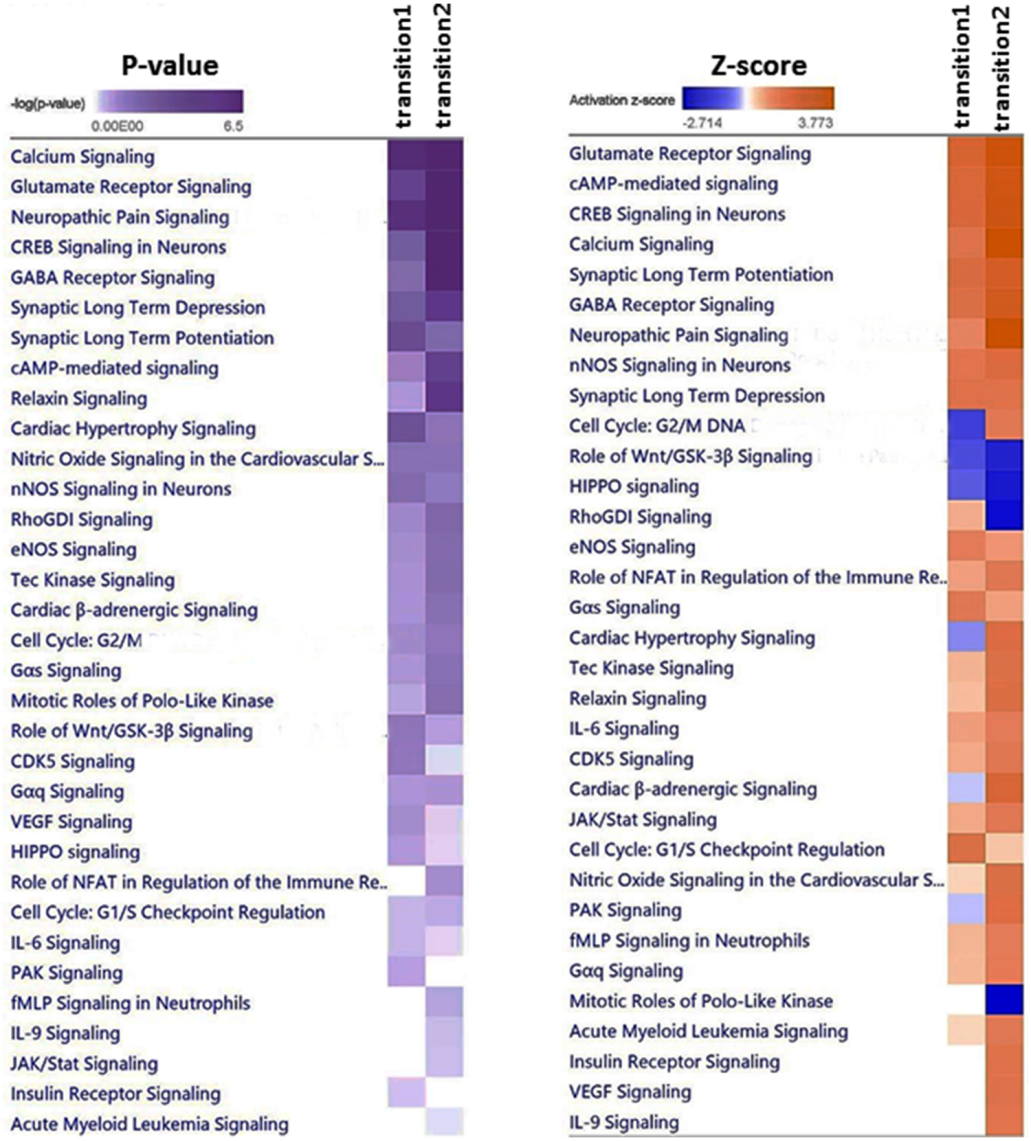
Heatmaps comparing activated canonical pathways between transition 1 (24 to 48 hpf) and transition 2 (48 to 96 hpf) based on p-value and activation z-score. The significance p-values were calculated by Fisher's exact test right-tailed. The significance indicates the probability of association of molecules from the dataset with the canonical pathway by random chance alone. A -log (p-value) cutoff was set to 1.3. The z-score algorithm was designed to reduce the chance that random data will generate significant predictions. IPA predicts that the canonical pathway is trending towards an increase when Z-scores ≥ 1.5. Blue indicates negative z-scores. IPA predicts that the canonical pathway is trending towards a decrease when Z-scores ≤ -1.5.

**Fig 6:**
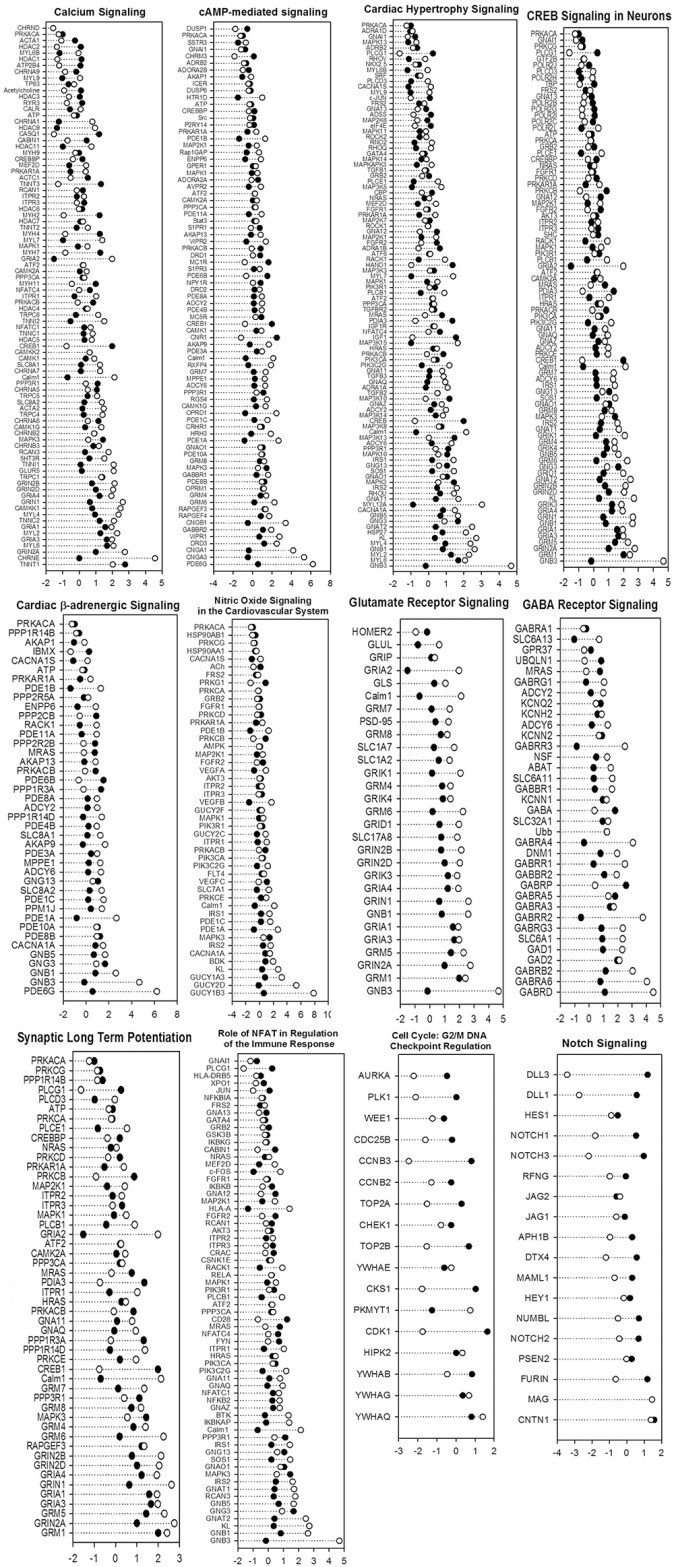
Corresponding genes to selected canonical pathways. Closed symbols represent significantly regulated genes (FDR < 0.05) for developmental transition 1 (from 24 to 48 hpf) and open symbols for transition 2 (from 48 to 96 hpf). The Y-axis plots gene symbols and the X-axis is the log2 fold change (FC).

**Fig 7:**
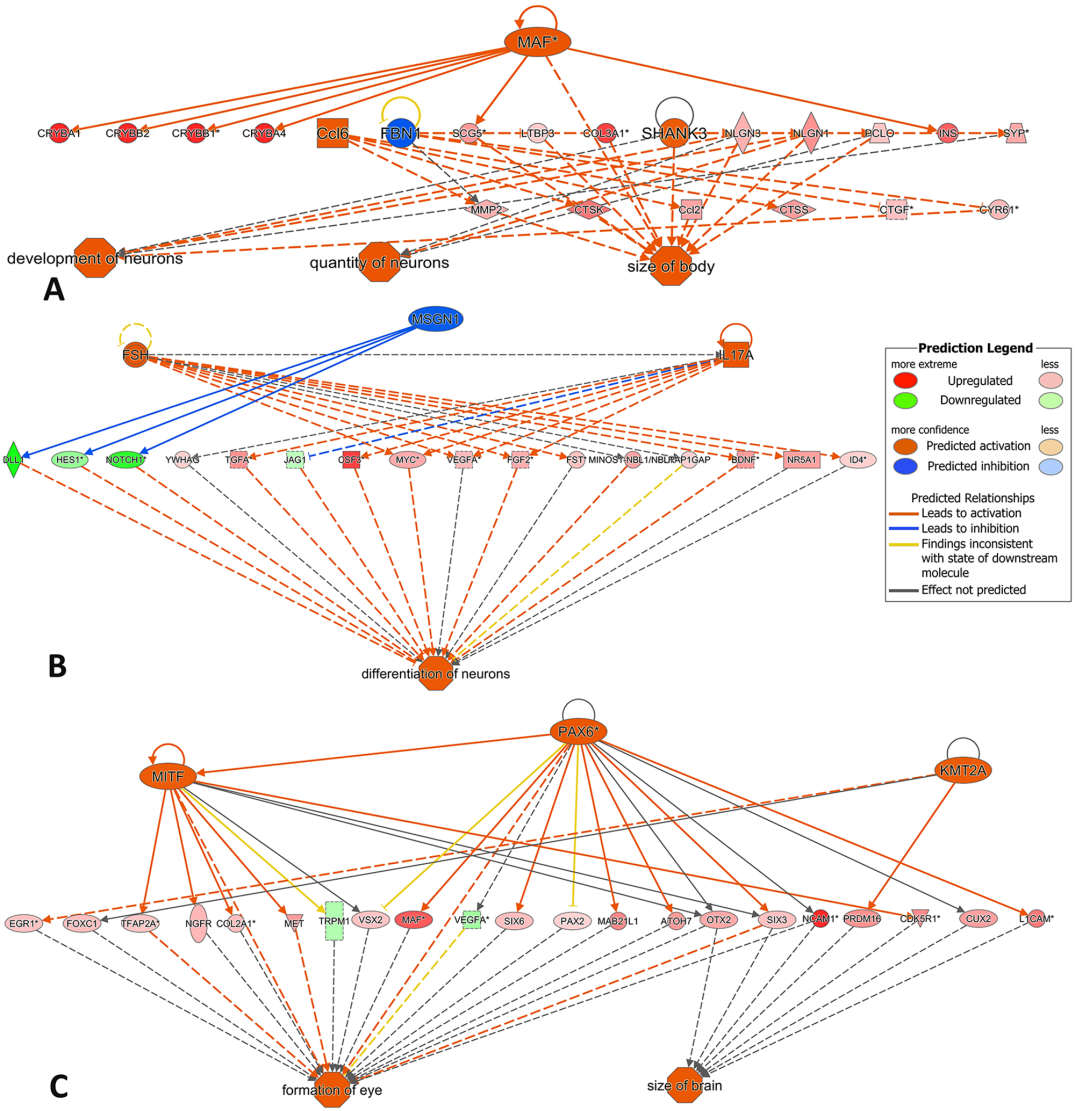
Predicted mechanisms through Ingenuity Pathway Analysis showing the expression of a number of developmental genes and regulators leading to: (A) development and quantity of neurons; (B) differentiation of neurons; (C) formation of eye and increasing size of brain.

To determine the commonalities in biological functions and canonical pathways during the two developmental transitions, comparative analysis was also carried out using IPA. The results indicated a proportion of biological functions common to both developmental transitions, although some functions were markedly more significant in one transition than the other (Fig 4). Cellular development, tissue development, embryonic development and endocrine system development were more significant in transition 1, whereas cell morphology, cell-to-cell signaling, gene expression, molecular transport, connective tissue development, carbohydrate metabolism, lipid metabolism, amino acid metabolism, cell cycle and urological system development were more significant in transition 2 (Fig 4), again reflecting a shift in the functions taking place during the two developmental transitions. Significant canonical pathways included calcium signaling, glutamate receptor signaling, cAMP response element-binding protein (CREB) signaling in neurons, gamma-aminobutyric acid (GABA) receptor signaling, protein kinase A signaling, synaptic long term depression and potentiation, cAMP-mediated signaling, relaxin signaling, cardiac hypertrophy signaling, and nitric oxide signaling in the cardiovascular system (Fig 5). Activation of the significant pathways was predicted by the z-score algorithm in IPA. The top activated common canonical pathways included glutamate receptor signaling, cAMP-mediated signaling, CREB signaling in neurons, calcium signaling, synaptic long term potentiation, GABA receptor signaling (Fig 5), which was consistent with the most significantly enriched terms related to neuronal systems by ToppGene (Table 2). The lists of DEGs responsible for the compared canonical pathways are reported in Fig 6 and S1 Table. Fig 7 predicts mechanisms showing the expression of a number of developmental genes and the upregulation of their key upstream regulators (i.e., MAF, FSH, IL17A, MITF, and PAX6) during the two developmental transitions; (A) development and quantity of neurons; (B) differentiation of neurons; (C) formation of eye and increasing size of brain. The top biological processes in both transitions included neurogenesis, generation of neurons, neuron differentiation, neuron development, and cell morphogenesis involved in differentiation (Table 2). The activated pathways were also consistent with signaling involved in development and function of the nervous system, including calcium signaling, glutamate receptor signaling, CREB signaling in neurons, GABA receptor signaling, and nNOS signaling (Fig 5).

## Discussion

The gene expression across critical developmental transitions is highly dynamic and has been shown to have long-term impacts on the normal development in mammalian and fish species [25–29]. In particular, the embryo-to-larval stage is a crucial period in the life of marine fish [30]. Marine fish larvae are generally less developed at hatching and have longer larval stages than freshwater fish [31]. During the embryo-to-larval phase, the mechanisms of organogenesis depend on the regulation of genes involved in multiple cellular processes, such as cell proliferation, differentiation, migration, as well as other biological pathways such as protein biosynthesis, and RNA processing [32]. The present study found a variety of biological functions that were differentially enriched in a temporal manner indicating the complexity of transcriptome regulation during the embryo-to-larval developmental transitions. General developmental biology (233 genes in cellular development, tissue development, organ development) was the most dominant biological function in transition 1 (Table 2), which is consistent with morphological and physiological observations during transition 1 (Fig 1). From 24 hpf to 48 hpf, the major morphological and physiological processes included somatic segmentation, tail elongation, initiation of heart beat and skeletal muscle contraction, formation of major veins, initiation of erythropoiesis and circulation, as well as retinal pigmentation (Fig 1). In contrast, transcripts related to metabolism functions were the most expressed in transition 2 (891 genes in metabolism in Table 2). In fish, the liver plays a central role in lipid metabolism, carbohydrate metabolism, and urological function [33], and all of these functions were more significantly enriched in transition 2 over transition 1 (Fig 4), consistent with terminal differentiation of the liver during later larval stages. In zebrafish, hepatocytes are identified by 36 hpf, and the functions of lipogenesis and xenobiotic metabolism are complete by 120 hpf [34]. While development of the liver in mahi-mahi has not been specifically examined in the present study, earlier studies have indicated the heart develops and differentiates from 48 hpf to 96 hpf comparable to zebrafish suggesting that the liver also likely develops at a similar time. In addition, histone deacetylase 3 (*hdac3*), a key transcription regulator specifically required for liver formation was only differentially expressed at 96 hpf but not 48 hpf in mahi-mahi, suggesting critical processes in liver formation in transition 2.

Consistent with studies in other marine fish species [30], the primary differentially expressed functional groups during the embryo-to-larval transition included nervous, muscular, and cardiovascular system development. The following discussion emphasizes the commonly activated canonical pathways involved in nervous, muscular and cardiovascular systems during the two developmental transitions and their responsive genes, including calcium signaling, glutamate receptor signaling, CREB signaling in neurons, GABA receptor signaling, nNOS signaling, cardiac β-adrenergic signaling, and nitric oxide signaling. These pathways are also likely to be specifically targeted by environmental stress or other changes during embryo-to-larva phase.

Similar to previous developmental transcriptome studies in common sole (*Solea solea*) [29] and Atlantic haddock (*Melanogrammus aeglefinus*) [3], nervous system development and function were the most significant enriched biological functions during embryo-to-larva transition (Table 2; Fig 4). This was consistent with the observations on the significant morphological changes and the development of the central (e.g. increased size of brain, and subdivided brain area; Fig 1) and sensory nervous system (e.g. pigmented retinal epithelium, and increased size of eyes; Fig 1) through the two developmental transitions.

Calcium plays a central role in signal transduction in cells thereby activating cellular growth and development [35]. External signals (e.g., growth factors, neurotransmitters, hormones) arriving at the cell activate plasma membrane receptors to initiate cell signaling pathways. One of the consequences of this signaling is increased intracellular calcium concentrations. The increase in cytosolic Ca^2+^ triggers a signaling cascade culminating in the regulation of transcription factors like NFAT, CREB and histone deacetylase (HDAC) which induce gene expression and other cellular events [36]. Ca^2+^ also plays a central role in muscle contraction by binding to the regulatory protein complex troponin [37]. In agreement with studies on embryonic development in rainbow trout *Oncorhynchus mykiss* [27], sea bream *Sparus auratus L*. [38], European sea bass *Dicentrarchus labrax* [39] and Atlantic haddock *Melanogrammus aeglefinus* [3], a group of muscle-related genes, encoding myosin light chain, troponin, and tropomyosin were upregulated during embryo-to-larva transition, reflecting the progressive development of muscle. In the present study, Calcium signaling was the most significant pathway changed (Fig 5; p-value = 6.4E-07 and 1.5E-10 for transition 1 and 2, respectively) with a number of corresponding genes (e.g. *tnnt1*, *tnnc2*, *grin2a*, *grin1*, *grin2b*, *grin2d*, *gria1*, *gria3*, *myl6*, *camk1g* etc.) significantly upregulated in both transitions (Fig 6). Proteins encoded by *tnnt1* and *tnnc2* are subunits of troponin, which regulates muscle contraction in response to fluctuations in intracellular calcium concentration. N-methyl-D-aspartate (NMDA) receptors are encoded by *grin* genes and are permeable to calcium ions [40]. Activation results in a calcium influx into post-synaptic cells, which continues the activation of several signaling cascades [41].

Glutamate receptor signaling pathway was the most activated pathway in both developmental transitions (Figs 5 and 6; z-sore = 2.9 and 3.6 for transition1 and 2, respectively). Glutamate has been recognized as the predominant excitatory neurotransmitter in the central nervous system (CNS), participating in a wide range of neural functions such as cell-to-cell signaling and interaction, synaptic plasticity, long-term potentiation and memory [42, 43]. Both metabotropic (*grm1*, *grm5*, *grm4*, *grm8*) and ionotropic glutamate receptors (*gria3*, *gria1*, *gria4*, *grin2d*, *grin2a*, *grin2b*, *grin1*) were among the most significantly upregulated in the two developmental transitions (Fig 6), similar to a previous study in early larvae of common sole (*Solea solea*) [29]. Both metabotropic and ionotropic glutamate receptors are involved in synaptic plasticity (S5 Fig). An increase in the number of ionotropic glutamate receptors on a postsynaptic cell may lead to long-term potentiation [44]. CREB signaling has also been documented in neuronal plasticity and long-term memory formation, and spatial memory in *Aplysia*, *Drosophila*, mice and rats [45]. CREB is also important for the proliferation and survival of neurons. Embryonic death was observed in mice lacking CREB, suggesting the critical role of CREB in normal embryonic development [46]. In both transition 1 and 2, CREB signaling was significantly activated in neurons via upregulation of glutamate receptors, and adenylate cyclase (S6 Fig). GABA receptor signaling was another one of the most activated pathways that begins with glutamate (Fig 5). The GABA receptors respond to the main inhibitory neurotransmitter in the vertebrate CNS [47]. In vertebrate brains, maintaining the excitation-inhibition (E/I) balance within neural circuits is important throughout life. The inhibitory GABAergic synaptic transmission plays a key role in the regulation of E/I balance [48]. A recent study reported that glutamate can control GABAergic synapses by activating GABAA receptor and destabilizing Ca^2+^ influx [49]. In the present study, a suite of genes coding metabotropic glutamate receptors and GABA receptors were significantly upregulated during the two transitions (Fig 6).

Nitric oxide (NO) is an important endogenous signaling molecule regulating synaptic signaling and plasticity [50]. nNOS signaling in neurons was a top ranked pathway during the two developmental transitions (Fig 5), with upregulation of a suite of responsive genes including glutamate ionotropic receptors, nitric oxide synthase, and protein kinase C. The expression of nNOS mRNA closely correlated with the neuronal differentiation pattern, involving the formation of the central and peripheral nervous system [51]. Additionally, nNOS protein is highly expressed in skeletal muscle [52]. nNOS controls muscle contractility through reacting with regulatory thiols on the sarcoplasmic reticulum [53]. nNOS activity is primarily regulated by Ca^2+^-dependent calpain degradation [54]. NO signaling in the cardiovascular system was also a top ranked pathway in the present study (Fig 5). Myocardial nNOS may regulate Ca^2+^ homeostasis through disrupting the expression of sarcoplasmic reticulum Ca^2+-^ATPase (SERCA), ryanodine receptor (RyR2), or Ca^2+^ channels (S7 Fig) [55]. In addition to NO signaling, β-adrenergic receptor (β-AR) signaling also controls cardiac contractility stimulation by the neurotransmitter norepinephrine (NE). In heart, β1-ARs are coupled to stimulatory G-proteins (Gαs). Stimulation of the receptor results in Gαs mediated activation of adenylate cyclase (AC) which then catalyzes the conversion of adenosine triphosphate (ATP) to cyclic adenosine monophosphate (cAMP). Higher cAMP levels lead to PKA activation, which regulates a number of Ca^+2^ cycling proteins like L-type Ca^+2^ channel, SERCA and (phospholamban) PLN (S8 Fig) [56].

With the advent of novel methods for deep RNA sequencing and sophisticated downstream bioinformatics pipelines, studies on developmental transcriptomes of non-model organism are gradually becoming affordable, enhancing the knowledge of the complex genetic control underling different process during organogenesis. The embryo-to-larval phase is a crucial period in the life of fish and very sensitive to environmental stress. Although model fish have been extensively used in the field of toxicogenomics, the detailed the embryonic transcriptomes of normal developing non-model fish is still rare. For example, Sørhus et al. showed a close match of the temporal gene expression pattern in between non-model and model fish [3]. In the present study, the normal developmental transcriptome of mahi-mahi, a pelagic fish species of global economic and ecological interests, was characterized for the first time in embryos and larvae. A genetic source for a number of significant canonical pathways and developmental genes is now available for this species which permits the delineation of mechanisms underlying physiological and morphological changes during the embryo-to-larval transition. In the context of environmental study, a comparative transcriptomic analysis comparing normal and stressed embryos will likely reveal novel mechanisms relating to abnormal development via disrupting the expression of developmental genes and molecular signaling pathways. Further refinement of comparative transcriptomes with other techniques, such as RNA-seq at single-embryo or tissue-level resolution, may allow more precise and exhaustive information to be gathered concerning genes and pathways associated with physiological processes involved in embryogenesis of fish that could be affected by non-chemical (e.g. global climate change) and chemical anthropogenic factors.

## Supporting information

S1 Fig PCA plot of transition 1 (a), transition 2 (b) and all three time points (c), and identity heat map (d). The percent variability attributed to the first two principal components is displayed on the X and Y-axes. Transition 1, 24 hpf to 48 hpf; Transition 2, 48hpf to 96 hpf.
(DOCX)

S2 Fig Molecular function networks enriched during transtion 1 (a) and transition 2 (b). (DOCX)

S3 Fig Cellular components enriched during transtion 1 (a) and transition 2 (b). (DOCX)

S4 Fig Biological process enriched during transtion 1 (a) and transition 2 (b). (DOCX)

S5 Fig Activation of Glutamate Receptor Signaling pathway during transition 1(A) and transition 2 (B). (DOCX)

S6 Fig Activation of CREB Signaling in Neurons pathway during transition 1 (A) and transition 2 (B). (DOCX)

S7 Fig Activation of Nitric oxide signaling in the cardiovascular system during transition 1 (A) and transition 2 (B). (DOCX)

S8 Fig Activation of Cardiac β-adrenergic Signaling pathway during transition 1 (A) and transition 2 (B). (DOCX)

S1 Table Top canonical pathways and log fold change of the corresponding genes during developmental transition 1 (24-48hpf) and transition 2 (48-96hpf). (DOCX)
