# Supplementary material for: Developmental transcriptomic analyses for mechanistic insights into critical pathways involved in embryogenesis of pelagic mahi-mahi (Coryphaena hippurus)
Source: PLoS One. 2017 Jul 10;12(7):e0180454. doi: 10.1371/journal.pone.0180454 (PMC5503239; doi:10.1371/journal.pone.0180454)
Supplement: S1 Fig — The percent variability attributed to the first two principal components is displayed on the X and Y-axes. Transition 1, 24 hpf to 48 hpf; Transition 2, 48hpf to 96 hpf. (DOCX) [file pone.0180454.s001.docx]

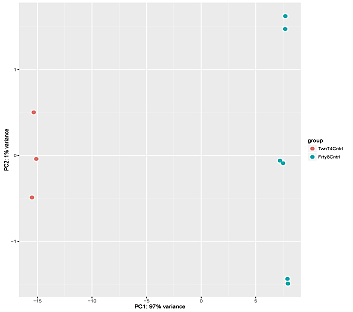

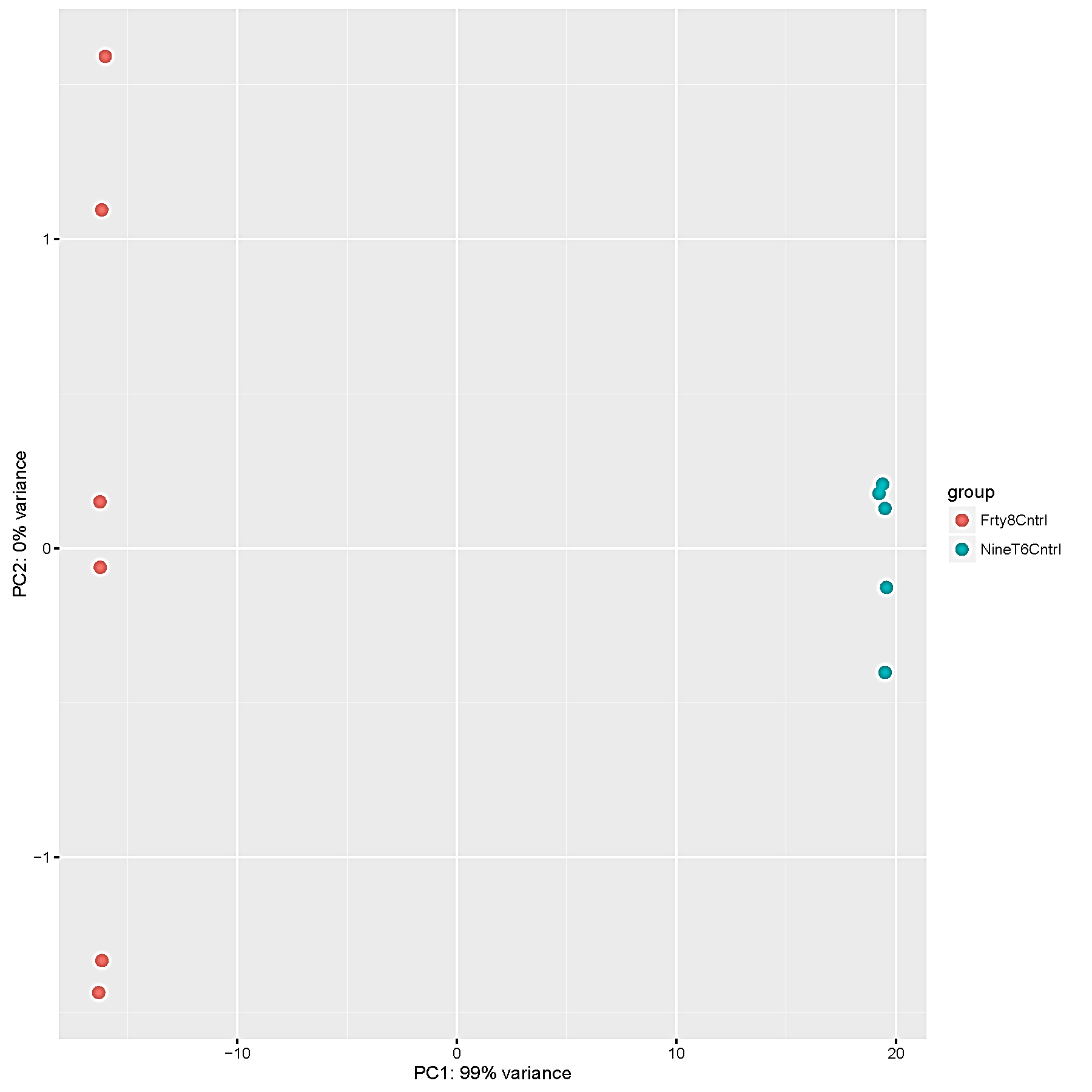

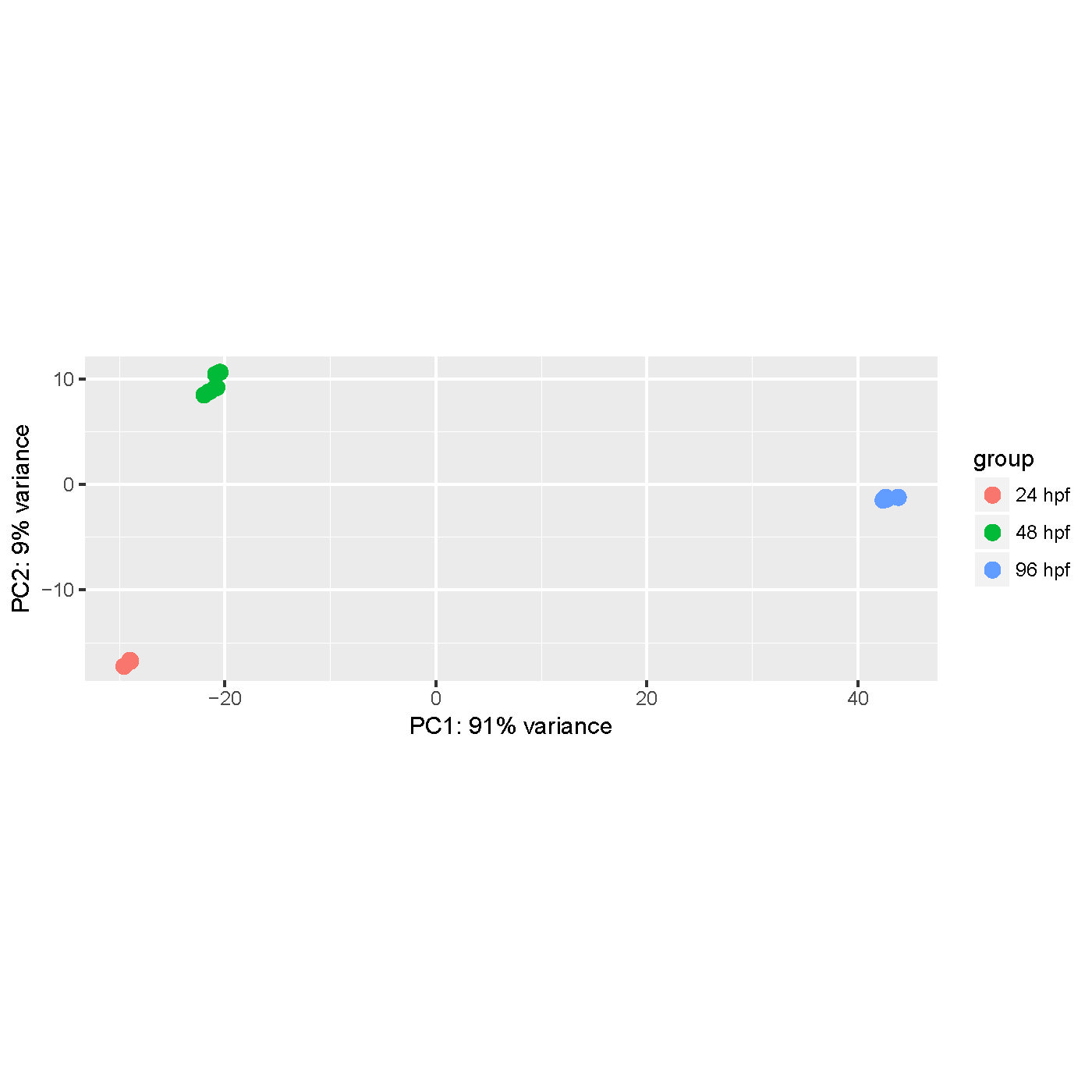

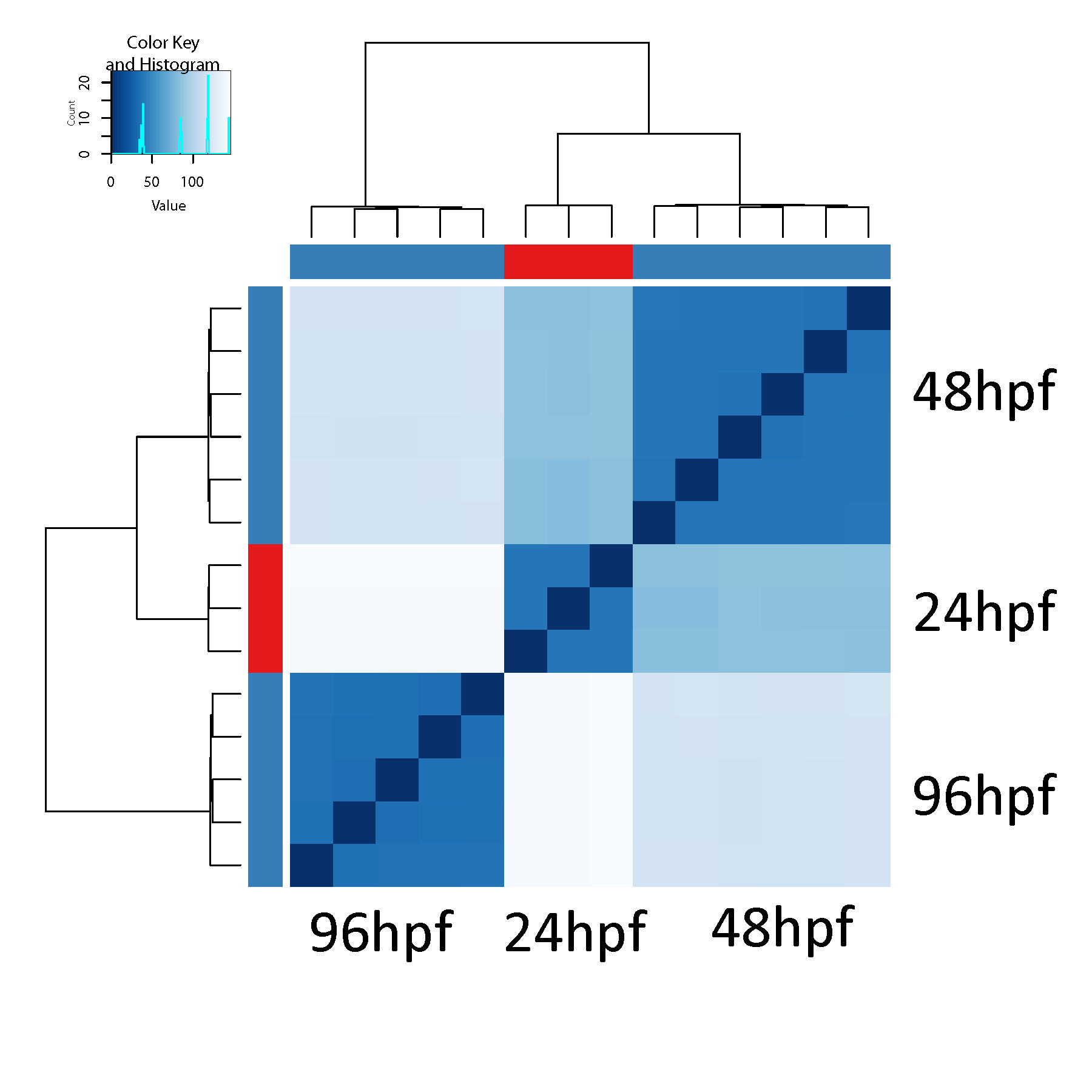


**d**

**c**

**b**

**a**

**S1 Fig.** PCA plot of transition 1 (a), transition 2 (b) and all three time points (c), and identity heat map (d). The percent variability attributed to the first two principal components is displayed on the X and Y-axes. Transition 1, 24 hpf to 48 hpf; Transition 2, 48hpf to 96 hpf.
