# Supplementary figures and images for: Developmental transcriptomic analyses for mechanistic insights into critical pathways involved in embryogenesis of pelagic mahi-mahi (Coryphaena hippurus)
Source: PLoS One. 2017 Jul 10;12(7):e0180454. doi: 10.1371/journal.pone.0180454 (PMC5503239; doi:10.1371/journal.pone.0180454)

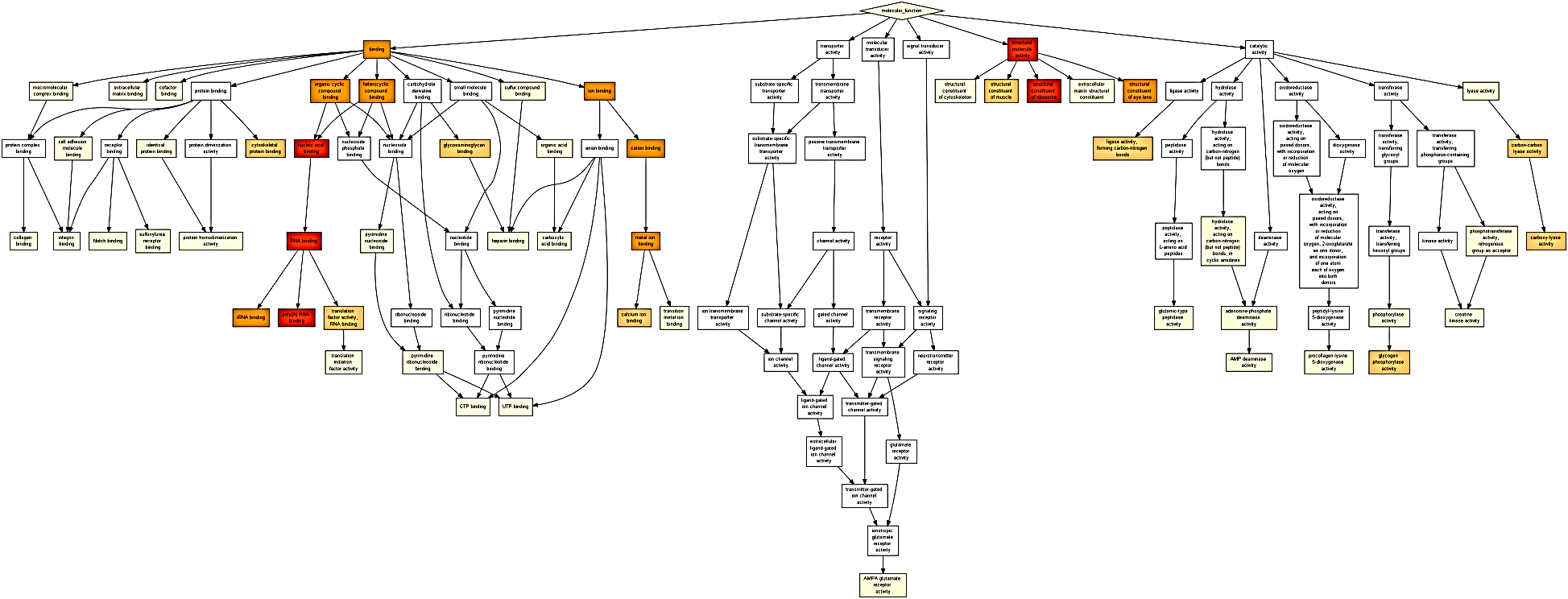

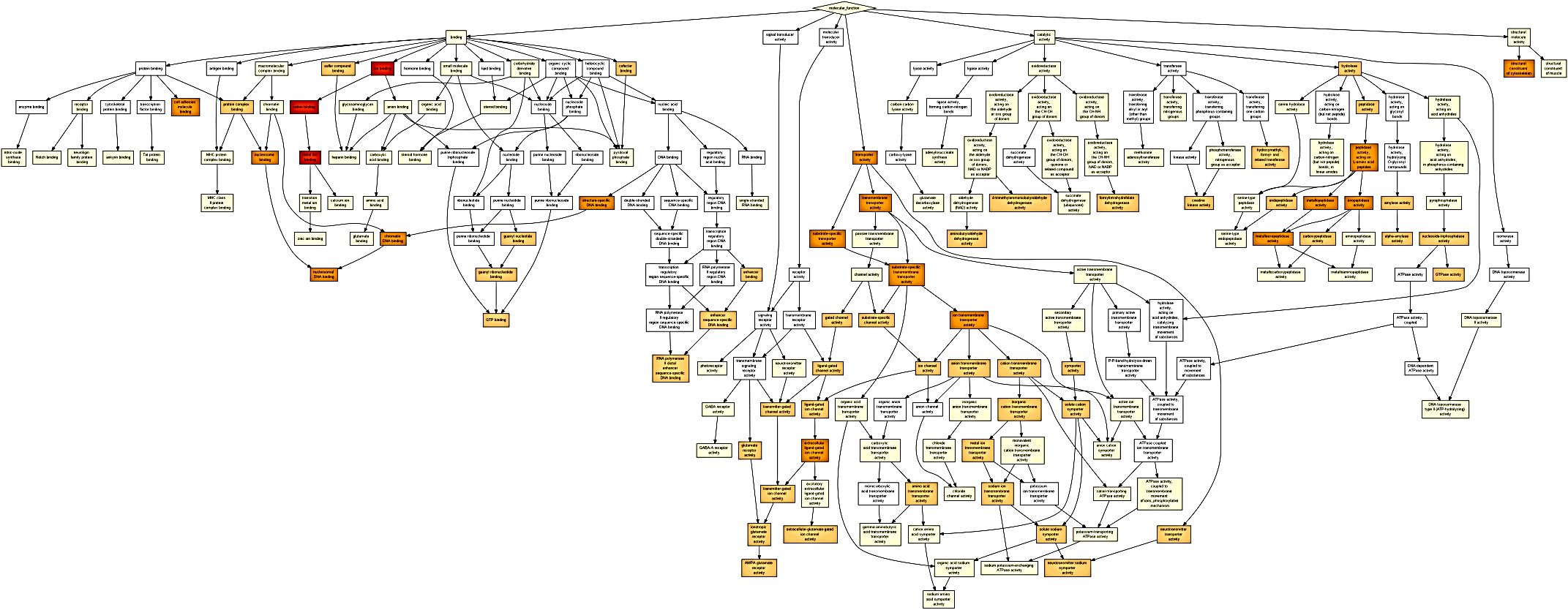


**B**

**A**

**S2 Fig.** Molecular function networks enriched during transtion 1 (a) and transition 2 (b).

Supplement: S2 Fig — (DOCX) [file pone.0180454.s002.docx]

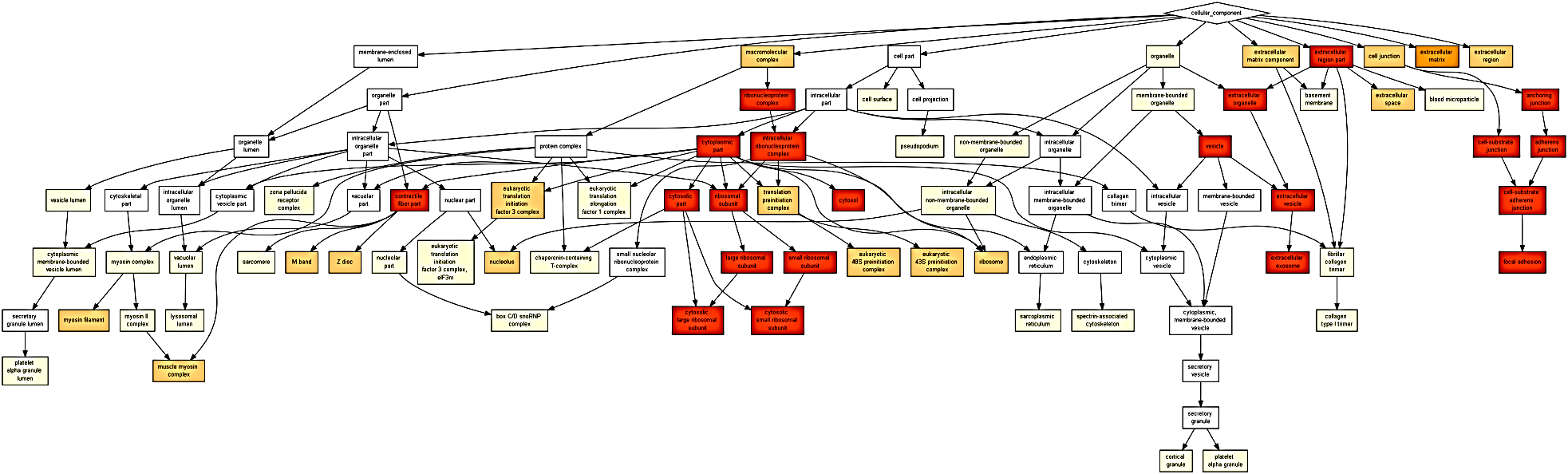

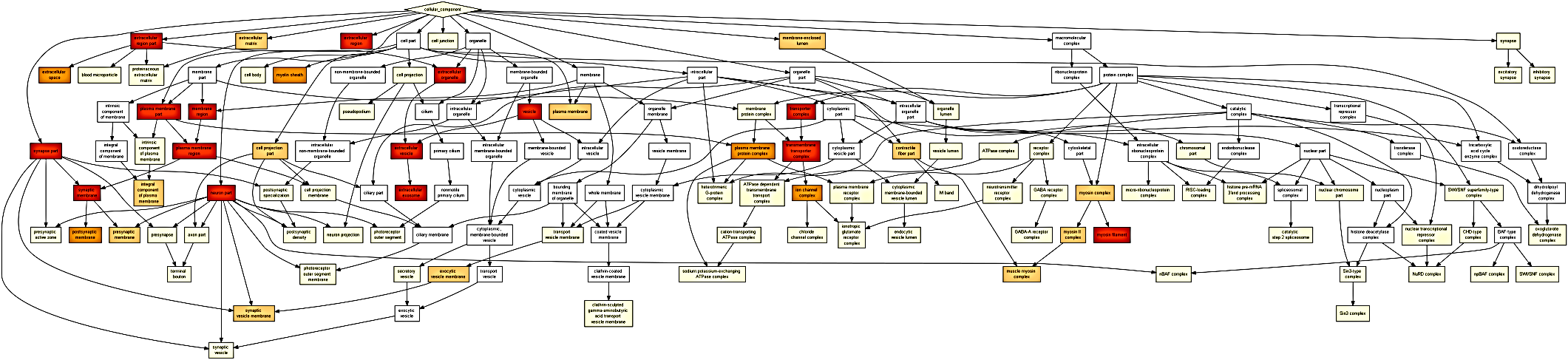


**A**

**B**

**S3 Fig.** Cellular components enriched during transtion 1 (a) and transition 2 (b).

Supplement: S3 Fig — (DOCX) [file pone.0180454.s003.docx]

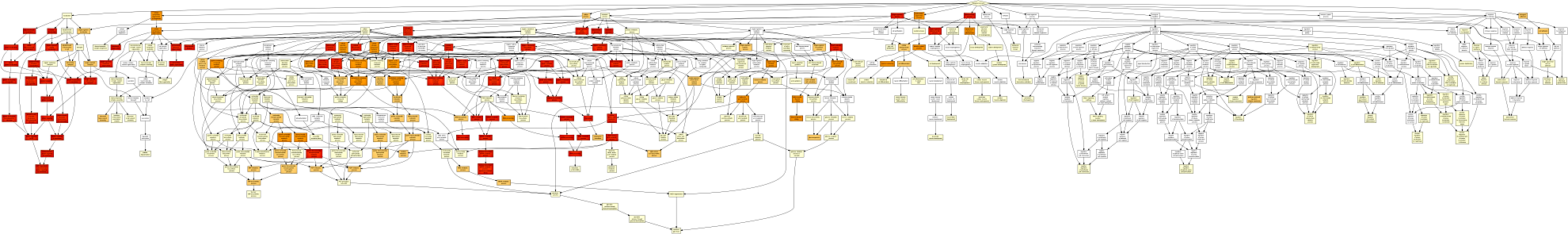




**S4 Fig.** Biological process enriched during transtion 1 (a) and transition 2 (b).

Supplement: S4 Fig — (DOCX) [file pone.0180454.s004.docx]

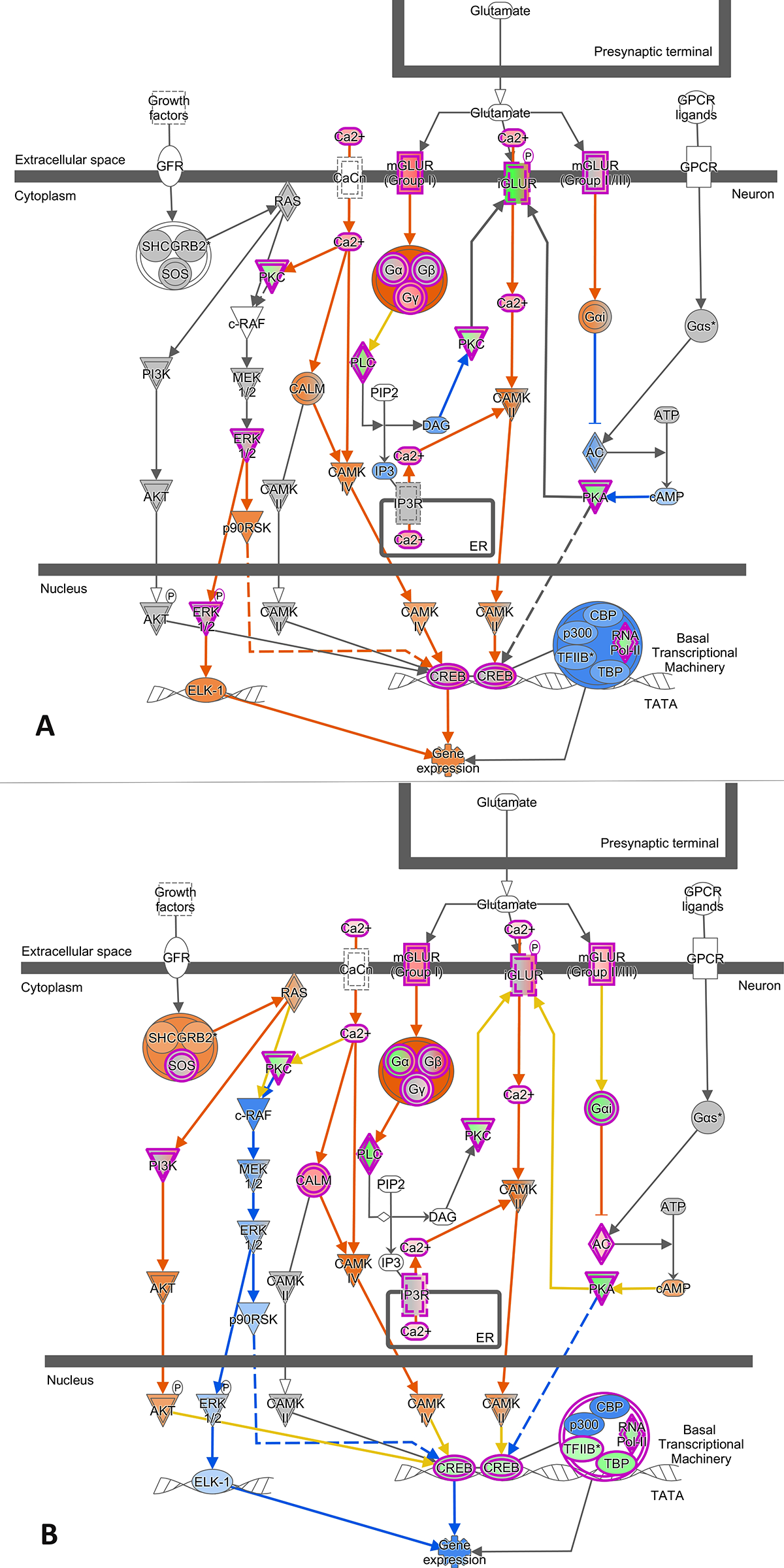


**S6 Fig.** Activation of CREB Signaling in Neurons pathway during transition 1 (A) and transition 2 (B).

Supplement: S6 Fig — (DOCX) [file pone.0180454.s006.docx]
