# Supplementary material for: Developmental transcriptomic analyses for mechanistic insights into critical pathways involved in embryogenesis of pelagic mahi-mahi (Coryphaena hippurus)
Source: PLoS One. 2017 Jul 10;12(7):e0180454. doi: 10.1371/journal.pone.0180454 (PMC5503239; doi:10.1371/journal.pone.0180454)
Supplement: S8 Fig — (DOCX) [file pone.0180454.s008.docx]

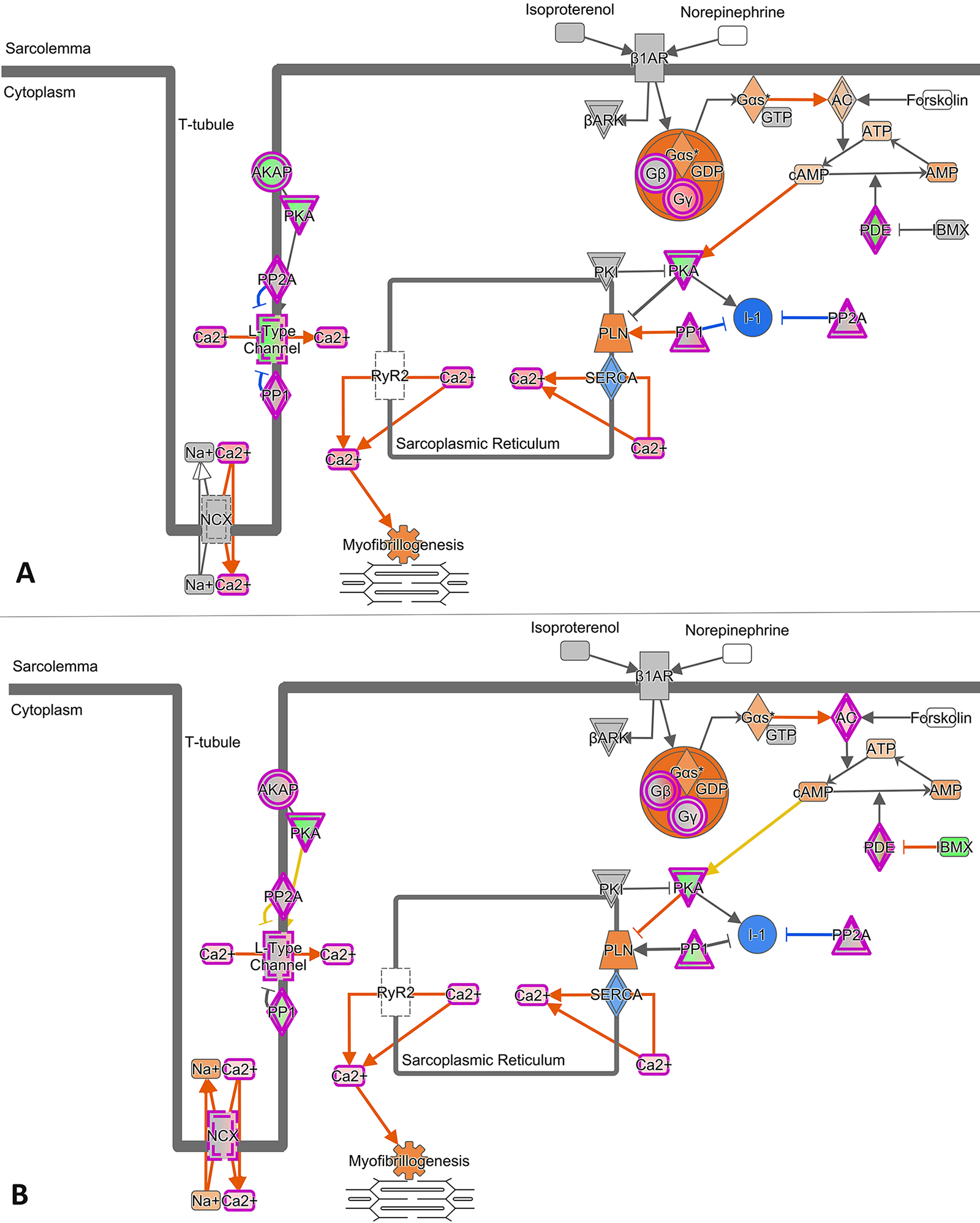


**S8 Fig.** Activation of Cardiac β-adrenergic Signaling pathway during transition 1 (A) and transition 2 (B).
