# Supplementary material for: Developmental transcriptomic analyses for mechanistic insights into critical pathways involved in embryogenesis of pelagic mahi-mahi (Coryphaena hippurus)
Source: PLoS One. 2017 Jul 10;12(7):e0180454. doi: 10.1371/journal.pone.0180454 (PMC5503239; doi:10.1371/journal.pone.0180454)
Supplement: S1 Table — (DOCX) [file pone.0180454.s009.docx]

**S1 Table** Top canonical pathways and log fold change of the corresponding genes during developmental transition 1 (24-48hpf) and transition 2 (48-96hpf).

| **Cardiac β-adrenergic Signaling network** | **Transition 1** | **Transition 2** | **Calcium Signaling network** | **Transition 1** | **Transition 2** | **cAMP-mediated signaling network** | **Transition 1** | **Transition 2** | **Genes in the Cardiac Hypertrophy Signaling network** | **Transition 1** | **Transition 2** |
| --- | --- | --- | --- | --- | --- | --- | --- | --- | --- | --- | --- |
| PDE6G | 0.6 | 6.2 | TNNT1 | 2.8 | 2.0 | PDE6G | 0.6 | 6.2 | GNB3 | -0.2 | 4.7 |
| GNB3 | -0.2 | 4.7 | CHRNE | N | 4.6 | CNGA3 | -0.5 | 5.3 | MYL6 | 1.7 | 2.1 |
| GNB1 | 0.8 | 2.6 | GRIN2A | 1.0 | 2.8 | CNGA1 | -0.4 | 4.2 | MYL2 | 1.3 | 2.3 |
| GNG3 | 1.7 | 0.9 | MYL6 | 1.7 | 2.1 | DRD3 | 1.2 | 2.5 | GNB1 | 0.8 | 2.6 |
| GNB5 | 0.7 | 1.7 | GRIA3 | 1.7 | 2.0 | VIPR1 | 0.7 | 2.8 | MYL4 | 1.0 | 2.4 |
| CACNA1A | 0.8 | 1.5 | MYL2 | 1.3 | 2.3 | GABBR2 | 1.1 | 1.9 | KL | 0.4 | 2.7 |
| PDE8B | 1.2 | 0.9 | GRIA1 | 1.6 | 1.9 | CNGB1 | -0.5 | 3.4 | HSP27 | 0.8 | 2.1 |
| PDE10A | 1.0 | 0.9 | TNNC2 | 1.2 | 2.1 | RAPGEF4 | 0.9 | 1.7 | GNAT2 | 0.4 | 2.5 |
| PDE1A | -0.8 | 2.7 | MYL4 | 1.0 | 2.4 | RAPGEF3 | 1.2 | 1.3 | GNG3 | 1.7 | 0.9 |
| PPM1J | 0.4 | 1.4 | CAMKK1 | 0.8 | 2.5 | GRM6 | 0.2 | 2.3 | GNB5 | 0.7 | 1.7 |
| PDE1C | 0.2 | 1.5 | GRIN1 | 0.6 | 2.6 | GRM4 | 0.8 | 1.4 | CACNA1A | 0.8 | 1.5 |
| SLC8A2 | 0.3 | 1.4 | GRIA4 | 1.2 | 1.9 | OPRM1 | 1.0 | 1.2 | MYL12A | -0.9 | 3.0 |
| GNG13 | 1.0 | 0.6 | GRIN2D | 1.0 | 2.0 | PDE8B | 1.2 | 0.9 | GNAT1 | 0.4 | 1.7 |
| ADCY6 | 0.2 | 1.3 | GRIN2B | 0.8 | 2.1 | GABBR1 | 0.4 | 1.6 | RHOU | 0.7 | 1.4 |
| MPPE1 | 0.2 | 1.2 | TRPC1 | 1.4 | 1.3 | MAPK3 | 1.4 | 0.5 | IRS2 | 0.5 | 1.6 |
| PDE3A | 0.4 | 1.0 | GLUR5 | 0.2 | 2.1 | GRM8 | 0.7 | 1.2 | MAPK3 | 1.4 | 0.5 |
| AKAP9 | -0.3 | 1.7 | TNNI1 | 0.1 | 2.1 | PDE10A | 1.0 | 0.9 | GNAO1 | 1.0 | 0.8 |
| SLC8A1 | 0.1 | 1.2 | 5HT3R | 0.6 | 1.5 | GNAO1 | 1.0 | 0.8 | SOS1 | 0.2 | 1.4 |
| PDE4B | 0.2 | 0.9 | RCAN3 | 0.4 | 1.8 | PDE1A | -0.8 | 2.7 | GNG13 | 1.0 | 0.6 |
| PPP1R14D | -0.3 | 1.4 | CHRNB3 | 0.9 | 1.2 | HRH3 | -0.1 | 1.9 | IRS1 | 0.2 | 1.4 |
| ADCY2 | 0.1 | 1.0 | MAPK3 | 1.4 | 0.5 | CRHR1 | 1.0 | 0.8 | MAPK10 | 1.1 | 0.6 |
| PDE8A | 0.1 | 1.0 | Ca2+ | 1.0 | 0.8 | PDE1C | 0.2 | 1.5 | PPP3R1 | 1.1 | 0.4 |
| PPP1R3A | 1.3 | -0.2 | CHRNB2 | 0.9 | 0.9 | OPRD1 | -0.8 | 2.5 | ADCY6 | 0.2 | 1.3 |
| PDE6B | 1.5 | -0.7 | CAMK1G | 0.4 | 1.3 | CAMK1G | 0.4 | 1.3 | MAP3K13 | 1.5 | 0.0 |
| PRKACB | 0.8 | -0.1 | CHRNA6 | 1.2 | 0.5 | RGS4 | 0.1 | 1.5 | Calm1 (includes others) | -0.7 | 2.1 |
| AKAP13 | -0.2 | 0.8 | TRPC4 | 0.3 | 1.4 | PPP3R1 | 1.1 | 0.4 | MAP3K8 | 0.7 | 0.6 |
| MRAS | 0.8 | -0.2 | ACTA2 | 0.2 | 1.5 | ADCY6 | 0.2 | 1.3 | CREB | 2.0 | -0.8 |
| PPP2R2B | 0.8 | -0.2 | SLC8A2 | 0.3 | 1.4 | MPPE1 | 0.2 | 1.2 | MAP3K14 | 0.4 | 0.8 |
| PDE11A | -0.4 | 0.9 | TRPC5 | 0.5 | 1.1 | GRM7 | 0.1 | 1.3 | ADCY2 | 0.1 | 1.0 |
| RACK1 | -0.6 | 0.9 | CHRNA5 | 1.1 | 0.5 | RXFP4 | -0.4 | 1.9 | GNAZ | 0.3 | 0.7 |
| PPP2CB | 0.9 | -0.6 | PPP3R1 | 1.1 | 0.4 | Calm1 | -0.7 | 2.1 | MAP3K10 | 1.2 | -0.2 |
| ENPP6 | -0.8 | 0.9 | Calm1 | -0.7 | 2.1 | PDE3A | 0.4 | 1.0 | TGFB2 | 0.5 | 0.5 |
| PPP2R5A | -0.2 | 0.2 | CHRNA7 | 0.1 | 1.3 | AKAP9 | -0.3 | 1.7 | ADRA1A | -0.2 | 1.1 |
| PDE1B | -1.4 | 1.3 | SLC8A1 | 0.1 | 1.2 | CNR1 | 2.5 | -1.2 | GNAQ | -0.1 | 0.9 |
| PRKAR1A | -0.5 | 0.4 | CAMK1 | 0.4 | 0.9 | CAMK1 | 0.4 | 0.9 | TGFB3 | 0.0 | 0.9 |
| ATP | -0.1 | -0.3 | CAMKK2 | 0.6 | 0.6 | CREB1 | 2.0 | -0.8 | GNA11 | 0.1 | 0.8 |
| CACNA1S | -1.1 | 0.1 | CREB1 | 2.0 | -0.8 | MC5R | 0.9 | 0.3 | PIK3C2G | -0.4 | 1.2 |
| IBMX | 0.3 | -1.4 | HDAC5 | 0.3 | 0.8 | PDE4B | 0.2 | 0.9 | PIK3CA | 0.5 | 0.3 |
| AKAP1 | -1.1 | -0.1 | TNNC1 | 0.3 | 0.8 | ADCY2 | 0.1 | 1.0 | PRKACB | 0.8 | -0.1 |
| PPP1R14B | -0.6 | -0.9 | NFATC1 | 0.3 | 0.7 | PDE8A | 0.1 | 1.0 | HRAS | 0.3 | 0.5 |
| PRKACA | -1.0 | -1.2 | TNNI2 | -0.5 | 1.5 | DRD2 | 0.2 | 0.7 | MAP3K15 | -1.0 | 1.6 |
| CREB Signaling in Neurons network | Transition 1 | Transition 2 | TRPC6 | -0.2 | 1.1 | NPY1R | 0.8 | 0.0 | IGF1 | 1.6 | -0.9 |
| GNB3 | -0.2 | 4.7 | HDAC4 | 0.4 | 0.5 | PDE6B | 1.5 | -0.7 | NFATC4 | 0.6 | 0.0 |
| GRM1 | 2.0 | 2.4 | PRKACB | 0.8 | -0.1 | S1PR3 | 0.1 | 0.7 | IGF1R | 0.3 | 0.3 |
| GRIN2A | 1.0 | 2.8 | ITPR1 | -0.3 | 1.0 | MC1R | 1.6 | -0.8 | PDIA3 | 1.3 | -0.7 |
| GRM5 | 1.4 | 2.3 | NFATC4 | 0.6 | 0.0 | DRD1 | 0.8 | 0.0 | MRAS | 0.8 | -0.2 |
| GRIA3 | 1.7 | 2.0 | MYH11 | 1.0 | -0.4 | PRKACB | 0.8 | -0.1 | TGFBR2 | 0.3 | 0.2 |
| GRIA1 | 1.6 | 1.9 | PPP3CA | 0.2 | 0.3 | VIPR2 | -0.6 | 1.4 | PPP3CA | 0.2 | 0.3 |
| GNB1 | 0.8 | 2.6 | CAMK2A | 0.0 | 0.5 | AKAP13 | -0.2 | 0.8 | ATF2 | 0.2 | 0.3 |
| GRIN1 | 0.6 | 2.6 | ATF2 | 0.2 | 0.3 | S1PR1 | 0.7 | -0.2 | PLCB1 | -0.4 | 0.9 |
| GRIA4 | 1.2 | 1.9 | GRIA2 | -1.5 | 2.0 | Stat3 | 0.2 | 0.4 | PIK3R1 | 0.4 | 0.1 |
| GRIK3 | 1.2 | 1.9 | MYH7 | 1.3 | -0.8 | PDE11A | -0.4 | 0.9 | MAPK1 | -0.1 | 0.5 |
| KL | 0.4 | 2.7 | MAPK1 | -0.1 | 0.5 | PPP3CA | 0.2 | 0.3 | MYL7 | -1.0 | 1.4 |
| GRIN2D | 1.0 | 2.0 | MYL7 | -1.0 | 1.4 | CAMK2A | 0.0 | 0.5 | MAP3K3 | 0.3 | 0.1 |
| GRIN2B | 0.8 | 2.1 | MYH4 | 1.2 | -0.8 | ATF2 | 0.2 | 0.3 | HAND1 | 1.4 | -1.0 |
| GNAT2 | 0.4 | 2.5 | TNNT2 | -0.2 | 0.5 | AVPR2 | -0.4 | 0.9 | RACK1 | -0.6 | 0.9 |
| GRID1 | 0.7 | 2.0 | HDAC7 | 0.1 | 0.2 | ADORA2A | 0.6 | -0.1 | ATF6 | 0.0 | 0.1 |
| GNG3 | 1.7 | 0.9 | MYH2 | 1.2 | -0.9 | MAPK1 | -0.1 | 0.5 | ADRA1B | 0.6 | -0.6 |
| GRM6 | 0.2 | 2.3 | HDAC6 | 0.2 | 0.0 | GPER1 | 0.0 | 0.3 | FGFR2 | 0.5 | -0.4 |
| GNB5 | 0.7 | 1.7 | ITPR3 | 0.3 | -0.1 | ENPP6 | -0.8 | 0.9 | MAP2K1 | -0.4 | 0.4 |
| GRIK4 | 0.9 | 1.4 | ITPR2 | -0.1 | 0.3 | Rap1GAP | -0.6 | 0.7 | GNA12 | 0.5 | -0.5 |
| GRM4 | 0.8 | 1.4 | RCAN1 | 0.2 | -0.2 | MAP2K1 | -0.4 | 0.4 | ROCK1 | 0.0 | 0.0 |
| GRIK1 | 0.2 | 2.1 | TNNT3 | 1.3 | -1.3 | PDE1B | -1.4 | 1.3 | MAP2K7 | 0.1 | -0.2 |
| GNAT1 | 0.4 | 1.7 | ACTC1 | 0.5 | -0.5 | PRKAR1A | -0.5 | 0.4 | PRKAR1A | -0.5 | 0.4 |
| IRS2 | 0.5 | 1.6 | PRKAR1A | -0.5 | 0.4 | P2RY14 | 0.1 | -0.3 | FGFR1 | 0.0 | -0.1 |
| MAPK3 | 1.4 | 0.5 | MEF2D | -0.6 | 0.4 | Src | 0.1 | -0.3 | MEF2D | -0.6 | 0.4 |
| GRM8 | 0.7 | 1.2 | CREBBP | 0.2 | -0.4 | CREBBP | 0.2 | -0.4 | NRAS | -0.2 | 0.0 |
| GNAO1 | 1.0 | 0.8 | MYH9 | 0.0 | -0.2 | ATP | -0.1 | -0.3 | CBP | 0.2 | -0.4 |
| SOS1 | 0.2 | 1.4 | HDAC11 | -1.0 | 0.7 | HTR1D | -1.5 | 1.0 | MAP3K5 | -0.9 | 0.7 |
| GNG13 | 1.0 | 0.6 | CABIN1 | 0.5 | -0.7 | DUSP6 | -0.2 | -0.4 | PLCE1 | -0.8 | 0.5 |
| IRS1 | 0.2 | 1.4 | CASQ1 | 1.2 | -1.5 | ICER | -0.3 | -0.4 | GSK3β | -0.1 | -0.2 |
| ADCY6 | 0.2 | 1.3 | HDAC9 | -1.3 | 1.0 | AKAP1 | -1.1 | -0.1 | GRB2 | 0.0 | -0.4 |
| GRM7 | 0.1 | 1.3 | CHRNA1 | -1.2 | 0.8 | ADORA2B | -0.4 | -1.0 | TGFB1 | -0.1 | -0.2 |
| Calm1 | -0.7 | 2.1 | ATP | -0.1 | -0.3 | ADRB2 | -0.6 | -0.9 | ATP | -0.1 | -0.3 |
| CREB1 | 2.0 | -0.8 | CALR | -0.6 | 0.1 | CHRM3 | 0.1 | -1.8 | MAPKAPK3 | -0.7 | 0.2 |
| PRKCE | 0.2 | 1.0 | RYR3 | 0.2 | -0.7 | GNAI1 | -0.8 | -1.2 | MAPK14 | -0.4 | -0.1 |
| ADCY2 | 0.1 | 1.0 | HDAC3 | 0.0 | -0.6 | SSTR3 | -1.5 | -0.7 | GATA4 | -0.2 | -0.3 |
| GNAZ | 0.3 | 0.7 | Acetylcholine | 0.1 | -0.9 | PRKACA | -1.0 | -1.2 | RHOQ | -0.7 | 0.1 |
| GNAQ | -0.1 | 0.9 | TP63 | -0.6 | -0.3 | DUSP1 | -0.5 | -1.8 | RND2 | -0.8 | 0.2 |
| GNA11 | 0.1 | 0.8 | MYL9 | -1.0 | 0.1 | CDK5 Signaling network | Transition 1 | Transition 2 | ROCK2 | -0.5 | -0.1 |
| PIK3C2G | -0.4 | 1.2 | CHRNA9 | -0.2 | -0.8 | P/Q CaCn | 0.8 | 1.5 | MAPK11 | -0.5 | -0.1 |
| PIK3CA | 0.5 | 0.3 | ATP2B4 | 0.1 | -1.2 | EGR1 | 0.8 | 1.2 | eIF4E | 0.1 | -0.8 |
| PRKACB | 0.8 | -0.1 | HDAC1 | 0.1 | -1.3 | MAPK3 | 1.4 | 0.5 | MAP2K6 | -0.1 | -0.6 |
| HRAS | 0.3 | 0.5 | MYL6B | -1.2 | 0.0 | BDNF | 0.6 | 1.3 | ADSS | 0.1 | -0.8 |
| ITPR1 | -0.3 | 1.0 | HDAC2 | 0.1 | -1.3 | PPM1J | 0.4 | 1.4 | GNA13 | -0.1 | -0.6 |
| PDIA3 | 1.3 | -0.7 | ACTA1 | -0.3 | -1.1 | CDK5R1 | 1.4 | 0.4 | FRS2 | -0.5 | -0.2 |
| MRAS | 0.8 | -0.2 | PRKACA | -1.0 | -1.2 | MAPK10 | 1.1 | 0.6 | c-JUN | 0.1 | -1.0 |
| CAMK2A | 0.0 | 0.5 | CHRND | -1.6 | -1.6 | ADCY6 | 0.2 | 1.3 | MYL9 | -1.0 | 0.1 |
| ATF2 | 0.2 | 0.3 | eNOS Signaling network | Transition 1 | Transition 2 | MAPK4 | 1.2 | 0.0 | CACNA1S | -1.1 | 0.1 |
| GRIA2 | -1.5 | 2.0 | GUCY1B3 | 0.6 | 7.9 | PPP1R14D | -0.3 | 1.4 | PLCD3 | -1.0 | 0.0 |
| PLCB1 | -0.4 | 0.9 | CNGA3 | -0.5 | 5.3 | ADCY2 | 0.1 | 1.0 | SRF | -0.6 | -0.5 |
| PIK3R1 | 0.4 | 0.1 | CHRNE | N/A | 4.6 | PPP1R3A | 1.3 | -0.2 | MYL6B | -1.2 | 0.0 |
| MAPK1 | -0.1 | 0.5 | GUCY1A3 | 0.8 | 3.2 | NGFR | 1.0 | 0.0 | NKX2.5 | -0.5 | -0.7 |
| RACK1 | -0.6 | 0.9 | CNGA1 | -0.4 | 4.2 | DRD1 | 0.8 | 0.0 | RHOV | -1.1 | -0.2 |
| SHC | 0.3 | 0.0 | KL | 0.4 | 2.7 | PRKACB | 0.8 | -0.1 | PLCG1 | 0.3 | -1.6 |
| ITPR3 | 0.3 | -0.1 | CNGB1 | -0.5 | 3.4 | HRAS | 0.3 | 0.5 | ADRB2 | -0.6 | -0.9 |
| ITPR2 | -0.1 | 0.3 | BDK | 0.8 | 2.0 | NGF | 0.7 | 0.0 | MAPK13 | -1.1 | -0.7 |
| AKT3 | 0.2 | 0.0 | IRS2 | 0.5 | 1.6 | MRAS | 0.8 | -0.2 | GNAI1 | -0.8 | -1.2 |
| FGFR2 | 0.5 | -0.4 | ESR1 | 1.0 | 0.8 | PPP2R2B | 0.8 | -0.2 | ADRA1D | -1.0 | -0.9 |
| MAP2K1 | -0.4 | 0.4 | IRS1 | 0.2 | 1.4 | MAPK1 | -0.1 | 0.5 | PRKACA | -1.0 | -1.2 |
| GNA12 | 0.5 | -0.5 | CHRNA5 | 1.1 | 0.5 | PPP2CB | 0.9 | -0.6 | **Cell Cycle: G2/M DNA Damage Checkpoint Regulation network** | **Transition 1** | **Transition 2** |
| PRKCB | 0.9 | -0.9 | ADCY6 | 0.2 | 1.3 | MAPK7 | 0.4 | -0.2 | YWHAQ | 0.8 | 1.4 |
| PRKAR1A | -0.5 | 0.4 | Calm1 | -0.7 | 2.1 | MEK1 | -0.4 | 0.4 | YWHAG | 0.3 | 0.7 |
| PRKCD | 0.2 | -0.4 | PRKCE | 0.2 | 1.0 | PPP2R5A | -0.2 | 0.2 | YWHAB | 0.8 | -0.5 |
| FGFR1 | 0.0 | -0.1 | ADCY2 | 0.1 | 1.0 | ITGA6 | 0.4 | -0.4 | HIPK2 | 0.0 | 0.3 |
| NRAS | -0.2 | 0.0 | PRKAA2 | 1.0 | 0.1 | PRKAR1A | -0.5 | 0.4 | CDK1 | 1.6 | -1.7 |
| CREBBP | 0.2 | -0.4 | CAT1 | -0.4 | 1.3 | NRAS | -0.2 | 0.0 | PKMYT1 | -1.3 | 0.7 |
| PLCE1 | -0.8 | 0.5 | VEGFC | 1.1 | -0.1 | Calpain-1 | -0.8 | 0.4 | CKS1 | 1.0 | -1.8 |
| GRB2 | 0.0 | -0.4 | FLT4 | 0.3 | 0.6 | LAMC1 | -0.2 | -0.2 | YWHAE | -0.6 | -0.2 |
| PRKCA | -0.2 | -0.2 | Gαq | -0.1 | 0.9 | NTRK2 | -1.1 | 0.7 | TOP2B | 0.7 | -1.5 |
| ATP | -0.1 | -0.3 | PIK3C2G | -0.4 | 1.2 | CDK5 | 0.2 | -0.6 | CHEK1 | -0.3 | -0.8 |
| POLR2L | -0.8 | 0.3 | PIK3CA | 0.5 | 0.3 | LAMA5 | -0.1 | -0.3 | TOP2A | 0.3 | -1.5 |
| POLR2C | 0.0 | -0.6 | PRKACB | 0.8 | -0.1 | ATP | -0.1 | -0.3 | CCNB2 | -0.3 | -1.3 |
| POLR2I | 0.1 | -0.8 | ITPR1 | -0.3 | 1.0 | ITGA2 | -0.7 | 0.3 | CCNB3 | 0.8 | -2.5 |
| POLR2G | 0.1 | -0.8 | PIK3R1 | 0.4 | 0.1 | MAPK14 | -0.4 | -0.1 | CDC25B | -0.2 | -1.6 |
| POLR2B | -0.1 | -0.7 | VEGFB | -1.5 | 1.7 | MAPK11 | -0.5 | -0.1 | WEE1 | -0.6 | -1.2 |
| GNA13 | -0.1 | -0.6 | ITPR3 | 0.3 | -0.1 | ITGB1 | -0.4 | -0.3 | PLK1 | 0.0 | -2.1 |
| FRS2 | -0.5 | -0.2 | ITPR2 | -0.1 | 0.3 | MAPK15 | -0.1 | -1.2 | AURKA | -0.5 | -2.2 |
| TBP | 0.0 | -0.9 | AKT3 | 0.2 | 0.0 | PPP1R14B | -0.6 | -0.9 | **Role of NFAT in Regulation of the Immune Response network** | **Transition 1** | **Transition 2** |
| POLR2H | -0.8 | -0.1 | HSPA9 | -0.7 | 0.8 | MAPK13 | -1.1 | -0.7 | GNB3 | -0.2 | 4.7 |
| PLCD3 | -1.0 | 0.0 | VEGFA | -0.8 | 0.9 | LAMA1 | 0.1 | -2.2 | GNB1 | 0.8 | 2.6 |
| POLR2J | -0.3 | -0.8 | FGFR2 | 0.5 | -0.4 | PRKACA | -1.0 | -1.2 | KL | 0.4 | 2.7 |
| GTF2B | -0.7 | -0.6 | PRKAA1 | 0.1 | -0.1 | **Glutamate Receptor Signaling network** | **Transition 1** | **Transition 2** | GNAT2 | 0.4 | 2.5 |
| PLCG1 | 0.3 | -1.6 | CHIP | 0.2 | -0.2 | GNB3 | -0.2 | 4.7 | GNG3 | 1.7 | 0.9 |
| PRKCG | -0.7 | -0.9 | PRKCB | 0.9 | -0.9 | GRM1 | 2.0 | 2.4 | GNB5 | 0.7 | 1.7 |
| GNAI1 | -0.8 | -1.2 | PRKAR1A | -0.5 | 0.4 | GRIN2A | 1.0 | 2.8 | RCAN3 | 0.4 | 1.8 |
| PRKACA | -1.0 | -1.2 | PRKCD | 0.2 | -0.4 | GRM5 | 1.4 | 2.3 | GNAT1 | 0.4 | 1.7 |
| **Gαs Signaling network** | **Transition 1** | **Transition 2** | FGFR1 | 0.0 | -0.1 | GRIA3 | 1.7 | 2.0 | IRS2 | 0.5 | 1.6 |
| CNGA3 | -0.5 | 5.3 | DNM2 | -0.2 | -0.1 | GRIA1 | 1.6 | 1.9 | MAPK3 | 1.4 | 0.5 |
| GNB3 | -0.2 | 4.7 | PDK1 | 0.0 | -0.3 | GNB1 | 0.8 | 2.6 | GNAO1 | 1.0 | 0.8 |
| CNGA1 | -0.4 | 4.2 | GRB2 | 0.0 | -0.4 | GRIN1 | 0.6 | 2.6 | SOS1 | 0.2 | 1.4 |
| VIPR1 | 0.7 | 2.8 | PRKCA | -0.2 | -0.2 | GRIA4 | 1.2 | 1.9 | GNG13 | 1.0 | 0.6 |
| GNB1 | 0.8 | 2.6 | ATP | -0.1 | -0.3 | GRIK3 | 1.2 | 1.9 | IRS1 | 0.2 | 1.4 |
| CNGB1 | -0.5 | 3.4 | PKG | 0.9 | -1.3 | GRIN2D | 1.0 | 2.0 | PPP3R1 | 1.1 | 0.4 |
| RAPGEF4 | 0.9 | 1.7 | FRS2 | -0.5 | -0.2 | GRIN2B | 0.8 | 2.1 | Calm1 | -0.7 | 2.1 |
| RAPGEF3 | 1.2 | 1.3 | Ach | 0.1 | -0.9 | SLC17A8 | 0.8 | 1.9 | IKBKAP | -0.1 | 1.4 |
| GNG3 | 1.7 | 0.9 | HSPA5 | -0.4 | -0.7 | GRID1 | 0.7 | 2.0 | BTK | -0.2 | 1.3 |
| HCK | 0.6 | 1.9 | CHRNA9 | -0.2 | -0.8 | GRM6 | 0.2 | 2.3 | GNAZ | 0.3 | 0.7 |
| GNB5 | 0.7 | 1.7 | LPAR4 | 0.1 | -1.2 | GRIK4 | 0.9 | 1.4 | NFKB2 | 0.3 | 0.7 |
| MAPK3 | 1.4 | 0.5 | HSP90AA1 | -0.6 | -0.5 | GRM4 | 0.8 | 1.4 | NFATC1 | 0.3 | 0.7 |
| CRHR1 | 1.0 | 0.8 | PLCG1 | 0.3 | -1.6 | GRIK1 | 0.2 | 2.1 | GNAQ | -0.1 | 0.9 |
| GNG13 | 1.0 | 0.6 | BDKRB1 | -0.1 | -1.3 | SLC1A2 | 0.6 | 1.3 | GNA11 | 0.1 | 0.8 |
| ADCY6 | 0.2 | 1.3 | PRKCG | -0.7 | -0.9 | SLC1A7 | 0.3 | 1.7 | PIK3C2G | -0.4 | 1.2 |
| CNR1 | 2.5 | -1.2 | HSP90AB1 | -0.6 | -1.0 | GRM8 | 0.7 | 1.2 | PIK3CA | 0.5 | 0.3 |
| CREB1 | 2.0 | -0.8 | PRKACA | -1.0 | -1.2 | Ca2+ | 1.0 | 0.8 | HRAS | 0.3 | 0.5 |
| MC5R | 0.9 | 0.3 | HSPA8 | -0.7 | -2.5 | PSD-95 | 0.4 | 1.3 | ITPR1 | -0.3 | 1.0 |
| ADCY2 | 0.1 | 1.0 | **GABA Receptor Signaling network** | **Transition 1** | **Transition 2** | GRM7 | 0.1 | 1.3 | FYN | 0.7 | 0.0 |
| MC1R | 1.6 | -0.8 | GABRD | 1.1 | 4.5 | Calm1 | -0.7 | 2.1 | NFATC4 | 0.6 | 0.0 |
| DRD1 | 0.8 | 0.0 | GABRA6 | 0.8 | 4.0 | GLS | 0.3 | 1.1 | MRAS | 0.8 | -0.2 |
| PRKACB | 0.8 | -0.1 | GABRB2 | 1.2 | 3.0 | GRIA2 | -1.5 | 2.0 | CD28 | 1.2 | -0.7 |
| VIPR2 | -0.6 | 1.4 | GAD2 | 2.0 | 2.1 | GRIP | 0.1 | 0.3 | PPP3CA | 0.2 | 0.3 |
| MRAS | 0.8 | -0.2 | GAD1 | 1.0 | 2.3 | GLUL | -0.8 | 0.6 | ATF2 | 0.2 | 0.3 |
| ATF2 | 0.2 | 0.3 | SLC6A1 | 0.9 | 2.3 | HOMER2 | -0.2 | -1.0 | PLCB1 | -0.4 | 0.9 |
| AVPR2 | -0.4 | 0.9 | GABRG3 | 0.9 | 2.4 | **Tec Kinase Signaling network** | **Transition 1** | **Transition 2** | PIK3R1 | 0.4 | 0.1 |
| ADORA2A | 0.6 | -0.1 | GABRR2 | -0.6 | 3.8 | GNB3 | -0.2 | 4.7 | MAPK1 | -0.1 | 0.5 |
| MAPK1 | -0.1 | 0.5 | GABRA3 | 1.5 | 1.7 | GNB1 | 0.8 | 2.6 | RELA | 0.2 | 0.2 |
| RACK1 | -0.6 | 0.9 | GABRA5 | 1.8 | 1.4 | KL | 0.4 | 2.7 | RACK1 | -0.6 | 0.9 |
| GPER1 | 0.0 | 0.3 | GABRP | 2.6 | 0.4 | GNAT2 | 0.4 | 2.5 | CSNK1E | 0.1 | 0.2 |
| MAP2K1 | -0.4 | 0.4 | GABBR2 | 1.1 | 1.9 | YES1 | 0.1 | 2.6 | CRAC | 0.4 | -0.2 |
| PRKAR1A | -0.5 | 0.4 | GABRR1 | 0.3 | 2.5 | ACTG1 | 1.6 | 1.0 | ITPR3 | 0.3 | -0.1 |
| SRC | 0.1 | -0.3 | DNM1 | 0.8 | 2.0 | GNG3 | 1.7 | 0.9 | ITPR2 | -0.1 | 0.3 |
| CREBBP | 0.2 | -0.4 | GABRA4 | -0.4 | 3.1 | HCK | 0.6 | 1.9 | AKT3 | 0.2 | 0.0 |
| ATP | -0.1 | -0.3 | Ubb | 1.2 | 1.3 | GNB5 | 0.7 | 1.7 | RCAN1 | 0.2 | -0.2 |
| RYR3 | 0.2 | -0.7 | SLC32A1 | 0.9 | 1.3 | GNAT1 | 0.4 | 1.7 | FGFR2 | 0.5 | -0.4 |
| ADORA2B | -0.4 | -1.0 | GABA | 1.8 | 0.4 | RHOU | 0.7 | 1.4 | HLA-A | -1.3 | 1.4 |
| ADRB2 | -0.6 | -0.9 | KCNN1 | 1.0 | 1.2 | IRS2 | 0.5 | 1.6 | MAP2K1 | -0.4 | 0.4 |
| CHRM3 | 0.1 | -1.8 | GABBR1 | 0.4 | 1.6 | STAT4 | -0.4 | 2.5 | GNA12 | 0.5 | -0.5 |
| PRKACA | -1.0 | -1.2 | SLC6A11 | 0.3 | 1.6 | STAT6 | 1.0 | 1.0 | IKBKB | 0.2 | -0.4 |
| **Relaxin Signaling network** | **Transition 1** | **Transition 2** | ABAT | 0.3 | 1.5 | GNAO1 | 1.0 | 0.8 | FGFR1 | 0.0 | -0.1 |
| GUCY1B3 | 0.6 | 7.9 | NSF | 0.5 | 1.3 | ACTA2 | 0.2 | 1.5 | c-FOS | -1.0 | 0.8 |
| PDE6G | 0.6 | 6.2 | GABRR3 | -0.9 | 2.5 | GNG13 | 1.0 | 0.6 | MEF2D | -0.6 | 0.4 |
| GUCY2D | -0.1 | 5.3 | KCNN2 | 0.9 | 0.7 | IRS1 | 0.2 | 1.4 | NRAS | -0.2 | 0.0 |
| GNB3 | -0.2 | 4.7 | ADCY6 | 0.2 | 1.3 | MAPK10 | 1.1 | 0.6 | CABIN1 | 0.5 | -0.7 |
| GUCY1A3 | 0.8 | 3.2 | KCNH2 | 0.6 | 0.9 | ITGA5 | 1.0 | 0.5 | IKBKG | -0.1 | -0.2 |
| GNB1 | 0.8 | 2.6 | KCNQ2 | 0.8 | 0.5 | VAV3 | 0.3 | 1.0 | GSK3B | -0.1 | -0.2 |
| KL | 0.4 | 2.7 | ADCY2 | 0.1 | 1.0 | FRK | 0.0 | 1.3 | GRB2 | 0.0 | -0.4 |
| GNAT2 | 0.4 | 2.5 | GABRG1 | -0.2 | 1.1 | PRKCE | 0.2 | 1.0 | GATA4 | -0.2 | -0.3 |
| GNG3 | 1.7 | 0.9 | MRAS | 0.8 | -0.2 | STAT1 | 0.0 | 1.2 | GNA13 | -0.1 | -0.6 |
| GNB5 | 0.7 | 1.7 | UBQLN1 | 0.8 | -0.3 | BTK | -0.2 | 1.3 | FRS2 | -0.5 | -0.2 |
| PDE8B | 1.2 | 0.9 | GPR37 | 0.1 | -0.3 | GNAZ | 0.3 | 0.7 | NFKBIA | -0.4 | -0.4 |
| GNAT1 | 0.4 | 1.7 | SLC6A13 | -1.0 | 0.7 | NFKB2 | 0.3 | 0.7 | JUN | 0.1 | -1.0 |
| IRS2 | 0.5 | 1.6 | GABRA1 | -0.2 | -0.4 | GNAQ | -0.1 | 0.9 | XPO1 | -0.3 | -0.8 |
| RLN3 | 0.4 | 1.6 | **Synaptic Long Term Potentiation network** | **Transition 1** | **Transition 2** | PTK2 | 0.1 | 0.8 | HLA-DRB5 | -0.5 | -0.8 |
| MAPK3 | 1.4 | 0.5 | GRM1 | 2.0 | 2.4 | GNA11 | 0.1 | 0.8 | PLCG1 | 0.3 | -1.6 |
| PDE10A | 1.0 | 0.9 | GRIN2A | 1.0 | 2.8 | PIK3C2G | -0.4 | 1.2 | GNAI1 | -0.8 | -1.2 |
| GNAO1 | 1.0 | 0.8 | GRM5 | 1.4 | 2.3 | PIK3CA | 0.5 | 0.3 | **Nitric Oxide Signaling in the Cardiovascular System network** | **Transition 1** | **Transition 2** |
| PDE1A | -0.8 | 2.7 | GRIA3 | 1.7 | 2.0 | FYN | 0.7 | 0.0 | GUCY1B3 | 0.6 | 7.9 |
| PDE1C | 0.2 | 1.5 | GRIA1 | 1.6 | 1.9 | MRAS | 0.8 | -0.2 | GUCY2D | -0.1 | 5.3 |
| GNG13 | 1.0 | 0.6 | GRIN1 | 0.6 | 2.6 | STAT3 | 0.2 | 0.4 | GUCY1A3 | 0.8 | 3.2 |
| IRS1 | 0.2 | 1.4 | GRIA4 | 1.2 | 1.9 | PIK3R1 | 0.4 | 0.1 | KL | 0.4 | 2.7 |
| ADCY6 | 0.2 | 1.3 | GRIN2D | 1.0 | 2.0 | RELA | 0.2 | 0.2 | BDK | 0.8 | 2.0 |
| MPPE1 | 0.2 | 1.2 | GRIN2B | 0.8 | 2.1 | STAT2 | -0.6 | 1.0 | CACNA1A | 0.8 | 1.5 |
| PDE3A | 0.4 | 1.0 | RAPGEF3 | 1.2 | 1.3 | RACK1 | -0.6 | 0.9 | IRS2 | 0.5 | 1.6 |
| CREB1 | 2.0 | -0.8 | GRM6 | 0.2 | 2.3 | JAK1 | 0.6 | -0.3 | MAPK3 | 1.4 | 0.5 |
| PDE4B | 0.2 | 0.9 | GRM4 | 0.8 | 1.4 | FGFR2 | 0.5 | -0.4 | PDE1A | -0.8 | 2.7 |
| ADCY2 | 0.1 | 1.0 | MAPK3 | 1.4 | 0.5 | ACTC1 | 0.5 | -0.5 | PDE1C | 0.2 | 1.5 |
| PDE8A | 0.1 | 1.0 | GRM8 | 0.7 | 1.2 | GNA12 | 0.5 | -0.5 | IRS1 | 0.2 | 1.4 |
| GNAZ | 0.3 | 0.7 | PPP3R1 | 1.1 | 0.4 | FGR | 1.1 | -1.2 | Calm1 (includes others) | -0.7 | 2.1 |
| NFKB2 | 0.3 | 0.7 | GRM7 | 0.1 | 1.3 | ACTB | 0.0 | -0.1 | PRKCE | 0.2 | 1.0 |
| GNAQ | -0.1 | 0.9 | Calm1 (includes others) | -0.7 | 2.1 | PRKCB | 0.9 | -0.9 | SLC7A1 | -0.4 | 1.3 |
| PDE6B | 1.5 | -0.7 | CREB1 | 2.0 | -0.8 | PRKCD | 0.2 | -0.4 | VEGFC | 1.1 | -0.1 |
| GNA11 | 0.1 | 0.8 | PRKCE | 0.2 | 1.0 | JAK2 | -0.1 | -0.1 | FLT4 | 0.3 | 0.6 |
| PIK3C2G | -0.4 | 1.2 | PPP1R14D | -0.3 | 1.4 | FGFR1 | 0.0 | -0.1 | PIK3C2G | -0.4 | 1.2 |
| PIK3CA | 0.5 | 0.3 | PPP1R3A | 1.3 | -0.2 | FOS | -1.0 | 0.8 | PIK3CA | 0.5 | 0.3 |
| PRKACB | 0.8 | -0.1 | GNAQ | -0.1 | 0.9 | SRC | 0.1 | -0.3 | PRKACB | 0.8 | -0.1 |
| MMP9 | 0.5 | 0.1 | GNA11 | 0.1 | 0.8 | GRB2 | 0.0 | -0.4 | ITPR1 | -0.3 | 1.0 |
| MRAS | 0.8 | -0.2 | PRKACB | 0.8 | -0.1 | PRKCA | -0.2 | -0.2 | GUCY2C | -0.4 | 0.9 |
| PDE11A | -0.4 | 0.9 | HRAS | 0.3 | 0.5 | PTK2B | -0.5 | 0.1 | PIK3R1 | 0.4 | 0.1 |
| GUCY2C | -0.4 | 0.9 | ITPR1 | -0.3 | 1.0 | ITGA2 | -0.7 | 0.3 | MAPK1 | -0.1 | 0.5 |
| PIK3R1 | 0.4 | 0.1 | PDIA3 | 1.3 | -0.7 | TNFSF10 | -1.4 | 0.9 | GUCY2F | N/A | 0.3 |
| MAPK1 | -0.1 | 0.5 | MRAS | 0.8 | -0.2 | RHOQ | -0.7 | 0.1 | VEGFB | -1.5 | 1.7 |
| RELA | 0.2 | 0.2 | PPP3CA | 0.2 | 0.3 | RND2 | -0.8 | 0.2 | ITPR3 | 0.3 | -0.1 |
| RACK1 | -0.6 | 0.9 | CAMK2A | 0.0 | 0.5 | TNF | -0.9 | 0.3 | ITPR2 | -0.1 | 0.3 |
| NPR1 | 0.0 | 0.4 | ATF2 | 0.2 | 0.3 | ITGB1 | -0.4 | -0.3 | AKT3 | 0.2 | 0.0 |
| GUCY2F | N/A | 0.3 | GRIA2 | -1.5 | 2.0 | GNA13 | -0.1 | -0.6 | VEGFA | -0.8 | 0.9 |
| AKT3 | 0.2 | 0.0 | PLCB1 | -0.4 | 0.9 | FRS2 | -0.5 | -0.2 | FGFR2 | 0.5 | -0.4 |
| VEGFA | -0.8 | 0.9 | MAPK1 | -0.1 | 0.5 | RHOV | -1.1 | -0.2 | MAP2K1 | -0.4 | 0.4 |
| ENPP6 | -0.8 | 0.9 | ITPR3 | 0.3 | -0.1 | ACTA1 | -0.3 | -1.1 | AMPK | 0.1 | -0.1 |
| FGFR2 | 0.5 | -0.4 | ITPR2 | -0.1 | 0.3 | PLCG1 | 0.3 | -1.6 | PRKCB | 0.9 | -0.9 |
| MEK1 | -0.4 | 0.4 | MAP2K1 | -0.4 | 0.4 | PRKCG | -0.7 | -0.9 | PDE1B | -1.4 | 1.3 |
| GNA12 | 0.5 | -0.5 | PRKCB | 0.9 | -0.9 | GNAI1 | -0.8 | -1.2 | PRKAR1A | -0.5 | 0.4 |
| PDE1B | -1.4 | 1.3 | PRKAR1A | -0.5 | 0.4 |  |  |  | PRKCD | 0.2 | -0.4 |
| PRKAR1A | -0.5 | 0.4 | PRKCD | 0.2 | -0.4 |  |  |  | FGFR1 | 0.0 | -0.1 |
| FGFR1 | 0.0 | -0.1 | NRAS | -0.2 | 0.0 |  |  |  | GRB2 | 0.0 | -0.4 |
| c-FOS | -1.0 | 0.8 | CREBBP | 0.2 | -0.4 |  |  |  | PRKCA | -0.2 | -0.2 |
| GRB2 | 0.0 | -0.4 | PLCE1 | -0.8 | 0.5 |  |  |  | PRKG1 | 0.9 | -1.3 |
| ATP | -0.1 | -0.3 | PRKCA | -0.2 | -0.2 |  |  |  | FRS2 | -0.5 | -0.2 |
| GNA13 | -0.1 | -0.6 | ATP | -0.1 | -0.3 |  |  |  | ACh | 0.1 | -0.9 |
| FRS2 | -0.5 | -0.2 | PLCD3 | -1.0 | 0.0 |  |  |  | CACNA1S | -1.1 | 0.1 |
| NFKBIA | -0.4 | -0.4 | PLCG1 | 0.3 | -1.6 |  |  |  | HSP90AA1 | -0.6 | -0.5 |
| c-JUN | 0.1 | -1.0 | PPP1R14B | -0.6 | -0.9 |  |  |  | PRKCG | -0.7 | -0.9 |
| GNAI1 | -0.8 | -1.2 | PRKCG | -0.7 | -0.9 |  |  |  | HSP90AB1 | -0.6 | -1.0 |
| PRKACA | -1.0 | -1.2 | PRKACA | -1.0 | -1.2 |  |  |  | PRKACA | -1.0 | -1.2 |
